# Decoding skin cancer classification: perspectives, insights, and advances through researchers’ lens

**DOI:** 10.1038/s41598-024-81961-3

**Published:** 2024-12-18

**Authors:** Amartya Ray, Sujan Sarkar, Friedhelm Schwenker, Ram Sarkar

**Affiliations:** 1 Department of Computer Science and Engineering, Jadavpur University, Kolkata, 700032 India; 2 Institute of Neural Information Processing, Ulm University, 89081 Ulm, Germany

**Keywords:** Skin cancer, Skin lesion classification, Machine learning, Deep learning, Medical image, Health care, Mathematics and computing

## Abstract

Skin cancer is a significant global health concern, with timely and accurate diagnosis playing a critical role in improving patient outcomes. In recent years, computer-aided diagnosis systems have emerged as powerful tools for automated skin cancer classification, revolutionizing the field of dermatology. This survey analyzes 107 research papers published over the last 18 years, providing a thorough evaluation of advancements in classification techniques, with a focus on the growing integration of computer vision and artificial intelligence (AI) in enhancing diagnostic accuracy and reliability. The paper begins by presenting an overview of the fundamental concepts of skin cancer, addressing underlying challenges in accurate classification, and highlighting the limitations of traditional diagnostic methods. Extensive examination is devoted to a range of datasets, including the HAM10000 and the ISIC archive, among others, commonly employed by researchers. The exploration then delves into machine learning techniques coupled with handcrafted features, emphasizing their inherent limitations. Subsequent sections provide a comprehensive investigation into deep learning-based approaches, encompassing convolutional neural networks, transfer learning, attention mechanisms, ensemble techniques, generative adversarial networks, vision transformers, and segmentation-guided classification strategies, detailing various architectures, tailored for skin lesion analysis. The survey also sheds light on the various hybrid and multimodal techniques employed for classification. By critically analyzing each approach and highlighting its limitations, this survey provides researchers with valuable insights into the latest advancements, trends, and gaps in skin cancer classification. Moreover, it offers clinicians practical knowledge on the integration of AI tools to enhance diagnostic decision-making processes. This comprehensive analysis aims to bridge the gap between research and clinical practice, serving as a guide for the AI community to further advance the state-of-the-art in skin cancer classification systems.

## Introduction

Cancer, as indicated by the World Health Organization (WHO), stands as a prominent global cause of mortality^1^. The WHO predicts that the total number of cancer diagnoses will be doubled over the next two decades^2^. Early detection of cancer plays a pivotal role in significantly mitigating mortality through effective treatment strategies. Skin cancer is one of the most common malignancies, with approximately 300,000 new cases diagnosed globally in 2018. According to the American Cancer Organization, over 132,000 cases of melanoma were reported in the United States in 2019 alone, with melanoma accounting for 4740 male and 2490 female deaths that year^3^. The American Cancer Organization further projected that in 2022, around 99,780 individuals in the United States would be diagnosed with melanoma, with an estimated 7650 succumbing to the disease^4^. More recent statistics from 2023 indicate that skin cancer represented 5.0% of all cancer diagnoses in the United States, with 97,160 new cases and 7990 deaths, accounting for 1.3% of total cancer-related fatalities^5^. This data highlights the widespread incidence and serious health impact of skin cancer on a global scale.

The prevalence of skin cancer has witnessed a steady increase over time, largely attributed to increased exposure to detrimental ultraviolet (UV) radiation from the sun^6^. Frequent and intense sun exposure can lead to sunburns, elevating the likelihood of developing skin cancer. Age appears to be a factor influencing the incidence, with older individuals exhibiting a higher susceptibility to the disease. Public health initiatives have concentrated on promoting awareness regarding sun protection practices, emphasizing the importance of measures such as sunscreen application and wearing protective clothing to mitigate the risks associated with excessive sun exposure. Additional significant causes include the presence of abnormal moles in the body and a family history of skin cancer^7^.

**Fig. 1:**
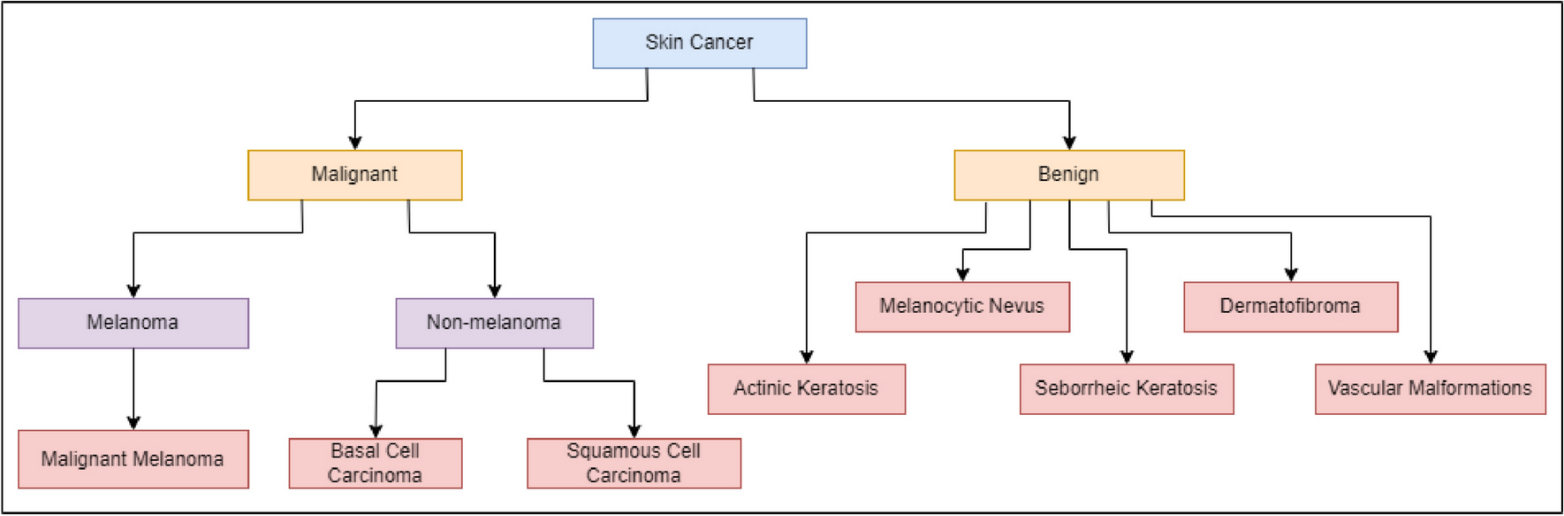
Types of skin cancer.

**Fig. 2:**
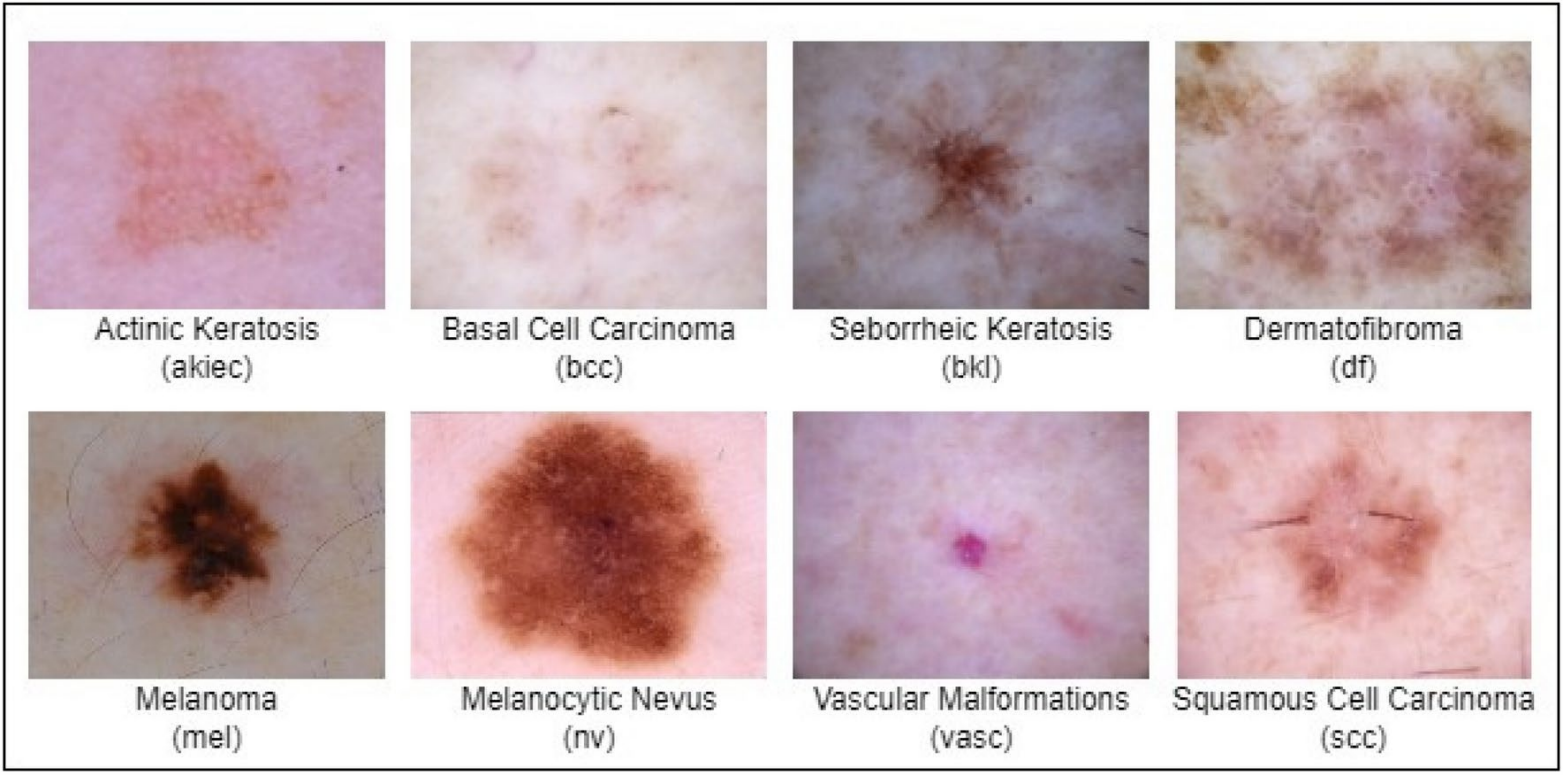
Sample images of various types of skin cancer.

Skin cancer is broadly categorized into two main types: melanoma and non-melanoma^8^. Figure 1 illustrates the categorization of skin cancer into different types and Fig. 2 presents images for each of these types. While basal cell carcinoma and squamous cell carcinoma constitute the most prevalent forms, they are not as harmful as melanoma^9^. Non-melanoma types primarily impact the middle and upper layers of the epidermis and exhibit a lower likelihood of spreading to other parts of the body^10^. On the other hand, melanoma originates in cells known as melanocytes, with the development typically initiated when these normally healthy cells undergo uncontrolled growth, forming a cancerous tumor. Any region of the skin is susceptible to melanoma, but it commonly manifests in areas with extensive exposure to sunlight, including the face, hands, and neck. Melanoma poses a higher risk of metastasis and contributes significantly to mortality related to skin cancer^11^. Early diagnosis is crucial for the effective cure of melanoma, as it spreads rapidly and can lead to a distressing and fatal outcome for the affected individual^12^.

### Challenges

The economic burden of skin cancer on healthcare systems is significant, given the expenses related to diagnosis, treatment, long-term care, and extensive medical training. In the United States alone, the financial impact is particularly striking, with treatment costs surpassing 8 billion dollars annually^13^. Traditional diagnosis of skin cancer primarily involves five methods: self-examination, visual inspection, mole mapping, dermoscopy, and skin biopsy. Accurate self-examination of skin cancer is highly challenging due to the striking resemblance between malignant and benign skin conditions. Individuals often struggle to distinguish between common skin irregularities and potential cancers, leading to missed diagnoses or unnecessary concerns. In clinical practice, dermatologists often rely on visual inspection to assess skin lesions based on the lesions’ structure and their evolving size or shape. However, this method is highly subjective and varies based on the experience and skill of the dermatologist. Additionally, relying solely on visual inspection can lead to unnecessary biopsies or missed diagnoses, especially for lesions with atypical features.

Mole mapping involves taking detailed photographs of a patient’s entire skin surface to track changes in moles or lesions over time^14^. Again, this relies on visual assessment and regular follow-up visits, which may delay diagnosis. Additionally, it may not capture changes in lesions that do not evolve in a predictable manner. Dermoscopy, a widely accepted imaging technique among dermatologists, involves magnifying the surface of a skin lesion for enhanced visibility during examination. However, its practice is limited to trained medical experts, relying heavily on their visual skills and experience^15^. The scarcity of well-trained professionals, attributed to the high costs and efforts involved in training, renders dermoscopy impractical for large-scale skin cancer detection. The gold standard for diagnosis, skin biopsy, involves surgically removing a sample of tissue from the suspected lesion for further analysis^16^. While highly accurate, biopsies are invasive, painful, and time-consuming. Additionally, biopsies are costly, and performing them unnecessarily on benign lesions increases healthcare expenses and patient discomfort.

These challenges highlight the necessity for novel and innovative approaches in visualizing and diagnosing skin cancer. Consequently, computer-aided diagnosis (CAD) systems have emerged as an effective technique for melanoma diagnosis. The evolution of CAD systems in dermatology initially focused on automating basic visual tasks like detecting shapes and borders in skin lesions. These early systems relied on hand-crafted features and rule-based algorithms but were limited in their ability to accurately differentiate between benign and malignant conditions due to the simplicity of the methods and the lack of large datasets. As digital imaging and dermoscopy became more common, CAD systems advanced by incorporating various image processing algorithms designed to assist dermatologists in identifying suspicious lesions. However, significant improvements came in the 2000s with the rise of machine learning (ML) models such as support vector machines (SVMs) and decision trees (DTs), which enhanced classification accuracy using manually extracted features like color, shape, and texture. The real breakthrough occurred in the 2010s with the advent of deep learning (DL), particularly convolutional neural networks (CNNs), which could automatically learn complex features from dermoscopic images. This leap in technology allowed CAD systems to perform at levels comparable to expert dermatologists, transforming them into essential tools for early skin cancer detection.

Such a system can offer a user-friendly environment particularly beneficial for less experienced dermatologists. The automated CAD system can aid dermatologists by reducing time, costs, and effort^17^. Additionally, it can serve as a valuable second opinion for doctors when dealing with complex melanoma cases. The mentioned challenges have spurred the focus of the artificial intelligence (AI) community towards the accurate and timely detection of skin cancer. Classifying skin cancer presents a complex challenge owing to the diverse and intricate nature of skin lesions. Numerous challenges impede the accurate and reliable classification of these lesions, rendering it a crucial area of investigation in medical imaging. A primary challenge stems from the subtle variations and resemblances among different skin lesions. The visual characteristics, such as color, texture, shape, and pattern, may overlap between benign and malignant lesions, complicating the differentiation between harmless moles and potentially cancerous ones. This inter-class similarity poses a significant hurdle for CAD systems. Another challenge emerges from the intra-class variability, referring to variations within the same class of skin lesions. Even within malignant lesions, considerable differences in appearance may exist. For instance, melanomas can manifest a diverse range of colors, shapes, and patterns, making it arduous to delineate clear boundaries between different types of lesions^18^. Additional challenges include the typically large size of individual skin lesion images, with only a restricted relevant portion indicating infection. Moreover, a deficiency in reliable annotated images further complicates the task of classification.

### Contributions

The main goal of this work is to review the progress made by researchers in the field of skin cancer classification using both ML and DL techniques. To the best of our knowledge, this survey includes most of the research papers published in this domain over the past 18 years. By consolidating the latest research, we seek to highlight both the potential and the challenges of integrating AI into clinical dermatology. This review is intended for a broad audience. For researchers in computer vision and medical imaging, this review acts as a valuable resource to understand the latest advancements, methodologies, and existing gaps in the field, helping shape future research directions. For clinicians, it offers insights into how AI tools can enhance their diagnostic processes, supporting early detection and improving decision-making. This survey can also be useful for healthcare policymakers aiming to understand the impact of AI-driven technologies in dermatology practices. Table 1 provides an overview of the contributions and limitations identified in some past survey papers. Additionally, our survey’s specific contributions are also outlined in this table.

**Table 1:** Comparison of existing survey papers with ours.

Authors	Year	Contributions	Limitations
Naeem et al.^19^	2020	It covers a wide range of techniques like handcrafted methods, CNNs, pre-trained models and ensemble-based approaches	It lacks an in-depth discussion of the individual techniques and does not articulate any noteworthy observations from the covered papers
Takkidin et al.^20^	2021	It employs a search strategy to include most papers between 2009 and 2020. It categorizes the papers into shallow and deep techniques	The detailed analysis of the specific techniques and their performance is limited due to the review format of the paper
Dildar et al.^21^	2021	It provides a comprehensive review of many papers based on different DL models like ANNs, CNNs, GANs etc.	Although it includes many papers, it lacks a detailed discussion of the models. Also, it does not include ML-based papers and does not mention future research directions
Wu et al.^22^	2022	Apart from discussing CNN-based techniques in detail, it highlights many key challenges and also suggests potential research avenues to address the challenges	It only focuses on CNN models across different datasets, but offers no discussion on ensemble-based approaches, GANs, hybrid strategies etc.
Naqvi et al.^23^	2023	It offers ample emphasis on the deep architectures of the methods discussed and throws light on the hardware resources required for each method	It categorizes papers broadly under the umbrella of DL but does not delve into specific techniques such as CNN-based or GAN-based etc., which could facilitate enhanced learning and understanding
Hasan et al.^24^	2023	It covers both conventional ML strategies and recent DL techniques including transfer learning, attention mechanisms, ensemble-based approaches etc.	Limited focus on the approaches and also does not mention any challenges or potential research gaps
Riaz et al.^25^	2023	It extensively discusses conventional CNN-based approaches, along with an in-depth analysis of transfer learning and federated learning methods. It also highlights significant research gaps and future directions	Limited exploration of advanced DL techniques, particularly those utilizing attention mechanisms, transformers, and meta-learning approaches
Ours	2024	Provides an in-depth discussion of ML, DL, hybrid and multimodal techniques with an emphasis on individual architectures, commonly used datasets, performance metrics, loss functions, open challenges and future research directions	Some possible future avenues of this survey are discussed in section “Future research directions”

### Organization

The subsequent sections of this paper are structured as follows: In section “Trends in skin cancer classification”, we outline the general trends in research within the domain of skin cancer classification throughout the years. Moving to section “Datasets used for skin cancer classification”, we explore the various datasets predominantly utilized by researchers in this field. In section “Strategies for skin cancer classification”, several computer vision-based strategies for skin cancer classification are discussed in detail. This section commences with an examination of the application of traditional ML techniques coupled with handcrafted features, followed by an in-depth overview of various DL strategies, including CNNs, generative adversarial networks (GANs), vision transformers (ViTs), and segmentation-guided classification methods. Hybrid and multimodal strategies for skin cancer classification have also been explored in this section. In sections “Performance metrics” and “Loss functions”, commonly used performance metrics and loss functions in the literature are discussed. Prevailing challenges in this field have been highlighted in section “Open challenges”, whereas, section “Future research directions” proposes potential solutions as avenues for future research. Finally, we present our concluding thoughts in section “Conclusion”.

## Trends in skin cancer classification

**Fig. 3:**
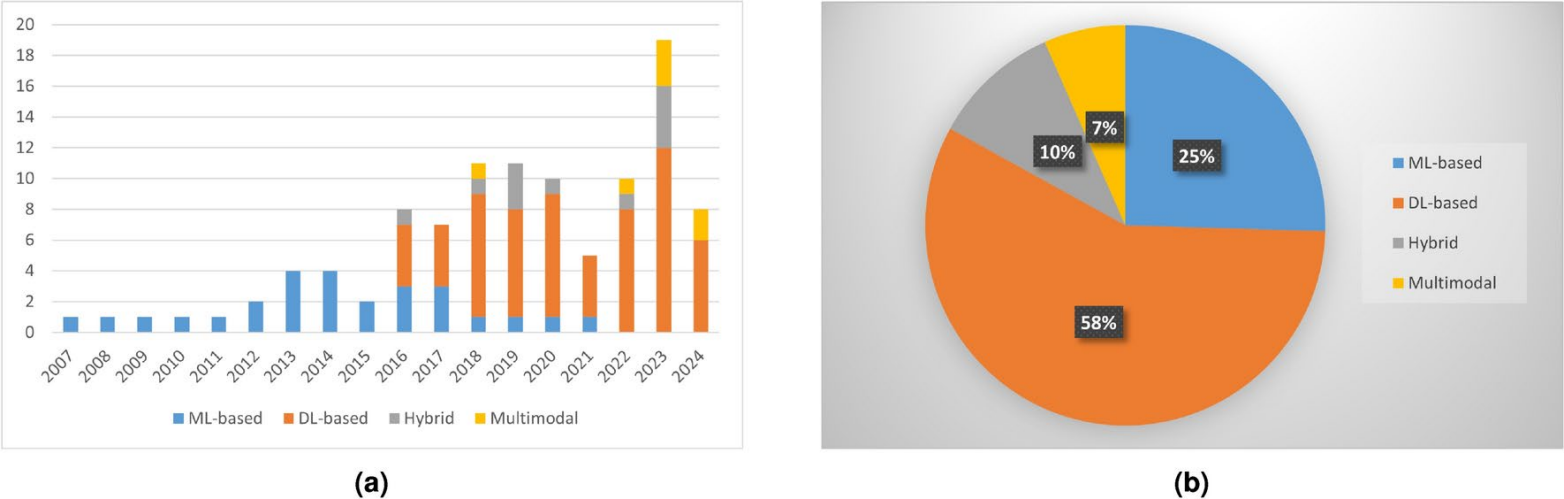
(**a**) Bar graph illustrating the distribution of research papers published over the past 18 years that leverage ML, DL, hybrid, and multimodal approaches. Note that this is not an exhaustive collection, but a subset selected for this survey. (**b**) Pie chart illustrating the percentage distribution of papers based on ML, DL, hybrid, and multimodal approaches.

The methodologies employed for skin cancer classification have emerged as pivotal components within CAD systems used for detecting skin diseases. This survey is centred on categorizing research papers on skin cancer classification published in the last two decades, predominantly emphasizing machine learning and deep learning approaches. In Fig. 3a, we present the distribution illustrating the count of research papers across these methodologies over almost 18 years, totalling 95 identified papers. We have utilized the search engines of Google Scholar, IEEE Xplore, Springer and ScienceDirect. Notably, various renowned journals in the domain of medical imaging and biomedical informatics, including IEEE Transactions on Medical Imaging, IEEE Journal of Biomedical and Health Informatics, IEEE Access, Scientific Reports, Computers in Biology and Medicine, Computer Methods and Programs in Biomedicine, Expert Systems with Applications, among others, have been considered.

As evident from Fig. 3a, with the advent of deep neural networks, there has been an escalating trend in the number of research articles published over the years, with a notable surge observed in 2018. More recently, there has also been a rise in the number of papers incorporating strategies involving a combination of machine learning and deep learning. Figure 3b depicts a pie chart illustrating the proportions of research papers using different computer vision-based strategies, with deep learning methodologies forming a significant portion of this survey. These insights offer valuable perspectives on the prevailing trends and prominence of various skin cancer classification strategies in the research landscape.

## Datasets used for skin cancer classification

Numerous computer vision-based systems aimed at diagnosing skin cancer have been proposed. However, effectively evaluating their diagnostic accuracy and validating predicted results necessitates a trustworthy compilation of dermoscopic images. Training neural networks for classifying skin lesions faces challenges due to the limited size and lack of diversity in existing datasets. Also, many researchers in the past have presented results based on their proprietary datasets, which might either lack public availability or fail to encompass real-world scenarios. Hence, the importance of having a standardized and reliable collection of dermoscopic images becomes very essential. To provide readers with a reference, this section delves into the several commonly used real-world datasets used for assessing proposed techniques in skin cancer classification. Table 2 gives the details of such datasets.

**Table 2:** Commonly used skin cancer classification datasets.

Name	Year	# Classes	# Images	Official split
HAM10000	2018	7	10,015	No
ISIC 2016	2016	2	1279	Yes (Train: 900, Test: 379)
ISIC 2017	2017	3	2750	Yes (Train: 2000, Valid: 150, Test-600)
ISIC 2018	2018	7	11,527	Yes (Train: 10015, Valid: 193, Test: 1512)
ISIC 2019	2019	8	33,569	Yes (Train: 25331, Test: 8238)
ISIC 2020	2020	2	44,108	Yes (Train: 33126, Test: 10982)
MED-NODE	2015	2	170	No
PH2	2013	3	200	No
DermIS	–	–	6588	No
DermQuest	1999	–	22,082	No
DermNet	1998	643	23,000	No
AtlasDerm	2000	7	1024	No
Dermofit	2014	10	1300	No

### HAM10000

The Human Against Machine dataset^26^, also known as HAM10000, is the latest publicly available skin lesions dataset comprising dermoscopic images sized at $450 \times 600$ pixels. It took two decades to assemble this dataset. These images primarily originated from Cliff Rosendahl’s skin cancer practice in Queensland, Australia, and the Dermatology Department of the Medical University of Vienna, Austria. Collected from diverse populations, various acquisition and cleaning methods were employed, along with the development of semi-automatic workflows, to address diversity issues. The dataset contains 10,015 images categorized into seven skin disease groups: 327 actinic keratosis and intraepithelial carcinoma images, 514 basal cell carcinoma images, 1099 benign keratosis images, 115 dermatofibroma images, 1113 melanoma images, 6705 melanocytic nevus images, and 142 vascular malformation images.

### ISIC archive

The ISIC archive^27^ is a collection of numerous skin cancer datasets. These datasets were introduced by the International Skin Imaging Collaboration (ISIC) at the International Symposium on Biomedical Imaging (ISBI) as individual challenges in order to improve melanoma diagnosis.

The ISIC 2016 challenge^28^ contains dermoscopic images segregated into training and testing subsets. The training set consists of 900 images, while the testing subset comprises 379 images. These images are categorized into two classes: malignant melanomas and benign nevi, with around 30.30% representing melanoma lesions and the rest belonging to benign nevi.

The ISIC 2017 challenge^29^ comprises 2750 images, belonging to three classes: melanoma, nevus and seborrheic keratosis. The dataset comprises 2000 training images, 150 validation images, and 600 testing images spanning all three classes. Notably, images in this dataset are captured by various imaging devices, leading to non-uniform resolutions. These images exhibit a range of resolutions, from smaller sizes like $300 \times 200$ pixels to larger resolutions such as $6000 \times 4000$ pixels. This diversity in image resolutions mirrors real-world scenarios, introducing complexity when analyzing images sourced from different devices.

The ISIC 2018 challenge^30^ contains 10,015 images belonging to seven skin disease categories for training, identical to the HAM10000 dataset. It contains 193 and 1512 additional images for validation and testing, respectively.

The ISIC 2019 challenge^31^ contains 25,331 images for training across eight skin lesion categories, including actinic keratosis, basal cell carcinoma, benign keratosis, dermatofibroma, melanoma, melanocytic nevus, vascular lesions and squamous cell carcinoma. The test dataset comprises 8239 images, including an outlier class not present in the training dataset. Additionally, the ISIC 2019 challenge provides metadata for images, such as patients’ sex, age, and affected area, necessitating new skin cancer diagnostic systems to identify and utilize these images. This dataset contains images with a range of sizes, from lower resolutions like $300 \times 200$ pixels to larger resolutions such as $6000 \times 4000$ pixels.

The ISIC 2020 challenge^32^ contains 33,126 and 10,982 dermoscopic images for training and testing, respectively. Each training image has a confirmed diagnosis (malignant or benign) and information about the patient’s age, sex, and lesion location. All diagnoses have been validated through histopathology for malignant cases, and for benign cases, confirmed via expert agreement, longitudinal follow-up, or histopathology.

### MED-NODE

The MED-NODE dataset^33^ consists of 170 dermoscopic images of skin lesions, out of which 70 images belong to melanoma and 100 images belong to nevi. These images have been collected from the digital archive of the Department of Dermatology of the University Medical Center, Groningen (UMCG).

### PH2

The PH2 database^34^ comprises a total of 200 dermoscopic images, consisting of 80 common nevi, 80 atypical nevi, and 40 melanomas. The images are sized at $768 \times 560$ pixels. Each image includes ground truth diagnosis, age, sex, and lesion location. This dataset is not publicly available due to privacy concerns and ethical restrictions. However, researchers can request access for research purposes through the Dermatology Service of Hospital Pedro Hispano, Matosinhos, Portugal.

### DermIS

The Dermatology Information System, commonly referred to as DermIS^35^, was collaboratively established by the University of Erlangen’s Department of Dermatology and the University of Heidelberg’s Department of Clinical Social Medicine. Comprising 6588 images, this dataset has recently been segmented into two sections: the dermatology online image atlas (DOIA) and the pediatric dermatology online image atlas (PeDOIA). The DOIA contains 3000 lesion images, encompassing around 600 dermatological diagnoses. It offers comprehensive dermoscopic images along with differential and provisional diagnoses, case reports, and detailed information covering nearly all categories of skin diseases.

### DermQuest

The publicly accessible DermQuest dataset^36^ comprised 22,082 dermoscopic images. Notably, among all dermoscopic datasets, only the DermQuest dataset included lesion tags for skin lesions, totalling 134 lesion tags for all images. The DermQuest dataset transitioned to Derm101 in 2018. However, this dataset was deactivated at the end of 2019.

### DermNet

The DermNet Skin Disease Atlas dataset, commonly known as DermNet^37^, originated in 1998 under the guidance of Dr. Thomas Habif in Portsmouth, New Hampshire. Comprising over 23,000 dermoscopic images, this database encompasses images depicting 643 distinct types of skin diseases. These diseases are categorized biologically into a two-tiered taxonomy. At the lower level, there are over 600 skin diseases organized with fine granularity. The top-level taxonomy comprises 23 different classes of skin diseases, such as connective tissue disease, benign tumors, eczema, melanomas, moles, and nevi, among others.

### AtlasDerm

The interactive Atlas of Dermoscopy dataset, known as AtlasDerm^207^, is a distinctive compilation combining a book and CD-ROM images featuring sample examples for training purposes. Initially developed as a diagnostic aid for physicians in identifying skin lesions and recognizing dermoscopic criteria linked to melanoma, the AtlasDerm dataset encompasses various cases of skin lesions, each accompanied by corresponding dermoscopic images. It comprises 5 images of actinic keratosis, 42 images of basal cell carcinoma, 70 images of benign keratosis, 20 images of dermatofibroma, 275 images of melanocytic nevus, 582 images of melanoma, and 30 images of vascular skin lesions.

### Dermofit image library

The Dermofit Image Library^38^ contains 1300 high-quality images of skin lesions captured under standardized color conditions, including internal color standards. It encompasses ten distinct classes of lesions: actinic keratosis, basal cell carcinoma, melanocytic nevus, seborrheic keratosis, squamous cell carcinoma, intraepithelial carcinoma, pyogenic granuloma, haemangioma, and dermatofibroma. Each image in this library possesses a gold standard diagnosis established through expert opinions, including dermatologists and dermatopathologists, ensuring precise labelling for algorithmic training purposes. Although not publicly available, researchers can access the Dermofit Image Library through Edinburgh Innovations, the technology transfer branch of the University of Edinburgh.

## Strategies for skin cancer classification

**Fig. 4:**
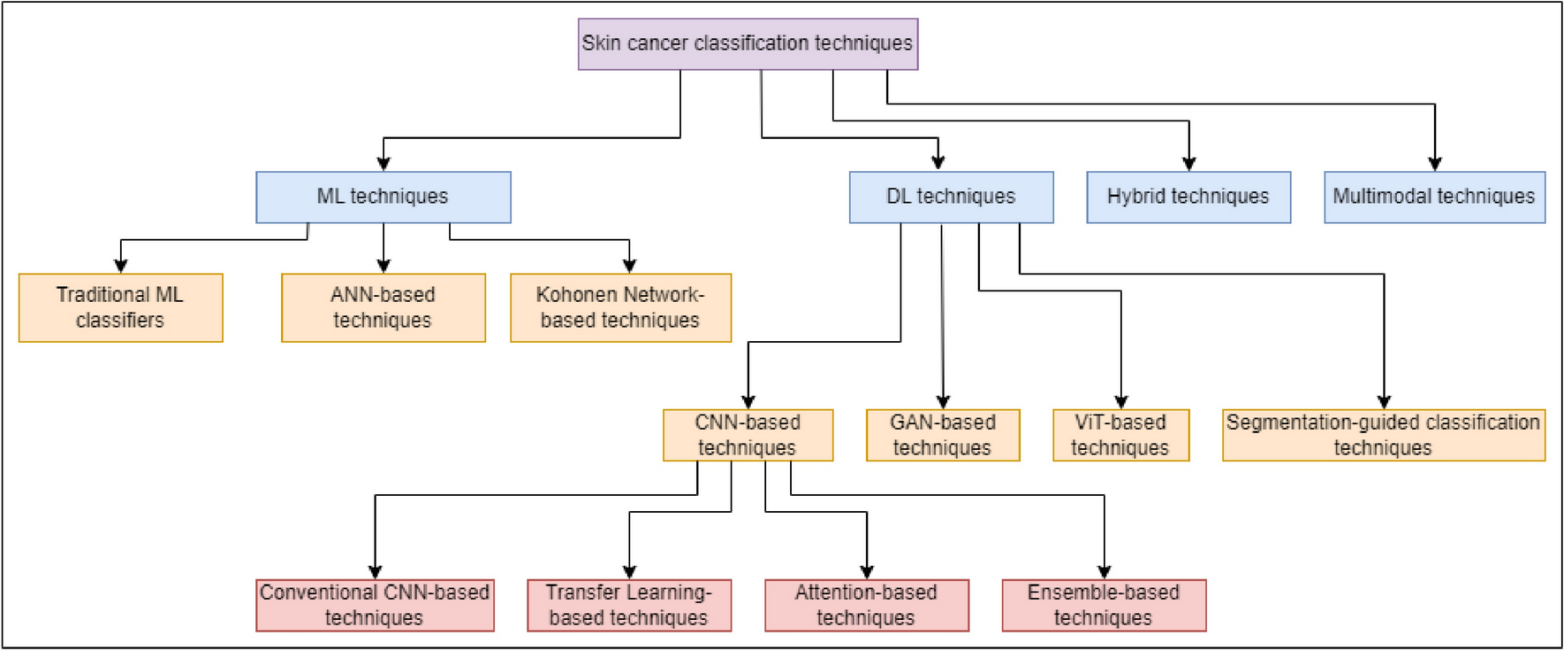
Taxonomy of different skin cancer classification strategies used in this survey.

In this section, we discuss in detail the various strategies based on computer vision that have been employed to classify skin cancer found within the literature. The taxonomy of the different strategies is shown in Fig. 4.

### Machine learning-based techniques

Classification of skin cancer can be considered a supervised learning problem which can be tackled using ML-based systems. Such systems traditionally rely on handcrafted features. These features are meticulously designed through image processing and feature engineering methods, capturing specific characteristics and patterns within skin lesions that signify different types of skin cancer. These manually crafted features are then input into diverse classifiers, including SVMs, random forests (RFs), k-nearest neighbors (k-NNs), or artificial neural networks (ANNs), facilitating the classification of skin cancer. Importantly, the process of extracting handcrafted features is less computationally demanding compared to training deep neural networks, rendering these techniques more adaptable to resource-constrained environments^39^. Nevertheless, a significant drawback of such systems is their dependence on the quality of manually crafted features and their generalizability across diverse datasets.

Traditional ML models often allow for greater interpretability, enabling clinicians to understand the reasoning behind predictions. This interpretability is crucial in clinical settings, as it fosters trust and facilitates informed decision-making based on model outputs. However, this interpretability comes at a cost. While traditional models like DTs offer clear insights into how predictions are made, they often struggle to capture the complex relationships inherent in the data, resulting in lower predictive performance compared to more sophisticated models, such as DL approaches. In contrast, DL models, although typically yielding higher accuracy, operate as “black boxes”, making it difficult to decipher the underlying rationale for their predictions. This lack of transparency can be problematic in medical contexts, where understanding the basis for a diagnosis is vital for patient care and compliance with ethical standards.

The challenge, therefore, lies in striking a balance between performance and interpretability. Clinicians may favor models that are easier to understand, even if they sacrifice some predictive power, while data scientists may lean towards models that offer higher accuracy but lack transparency. As highlighted in recent literature, addressing this trade-off is crucial for the successful integration of machine learning systems in healthcare, where both precision and trust are paramount. In this section, we offer an overview of the existing ML-based approaches outlined in the literature, summarizing their employed feature extraction techniques, classifiers, and performance outcomes across the datasets utilized.

#### Traditional machine learning classifier-based techniques

This segment delves into the different methodologies for skin cancer classification using handcrafted features in conjunction with ML classifiers. A comprehensive comparison of skin cancer classification methods using ML classifiers is discussed in Table 3.

Jørgensen et al.^40^ explored the collective utilization of various optical coherence tomography (OCT) features extracted from images of basal cell carcinoma and actinic keratosis. They evaluated the diagnostic accuracy of these combined features through an ML approach. The results of the ML analysis indicated that the use of a multitude of features led to an accuracy of 77%. Zortea et al.^41^ proposed a method aiming to capture local spatial information by utilizing local binary pattern histograms (LBPH)^42^ from the images. The extracted features underwent clustering through k-means clustering and were then input into an SVM for the classification of images as malignant or benign. Ballerini et al.^43^ introduced a skin cancer classification system that integrates both color and texture features. They employed a hierarchical k-NN classifier for the classification task. Color features were represented by the mean colors $\{\mu = (\mu R, \mu G, \mu B)\}$ of the lesion along with their covariance matrices. Texture features were extracted from generalized co-occurrence matrices (GCM)^44^. From each GCM, they derived 12 texture features, including energy, contrast, correlation, entropy, homogeneity, inverse difference moment, cluster shade, cluster prominence, max probability, autocorrelation, dissimilarity, and variance.

Mhaske et al.^45^ employed the 2D wavelet technique^46^ to generate 96 features from the images. Subsequently, these features were utilized by an SVM to classify the images as either malignant or benign. Maurya et al.^47^ proposed a skin cancer classification system, employing the gray level co-occurrence matrix (GLCM)^48^ as a feature extraction technique. Initially, the RGB image underwent conversion into a grayscale image, serving as input for GLCM. The computation by GLCM focused on the frequency of specific gray levels reappearing at different positions in the image. Feature extraction via GLCM mapped probabilities of gray level co-occurrence at various angular positions, relying on spatial relationships between different pixel combinations. Features like autocorrelation, contrast, energy, entropy, and homogeneity were then extracted from the matrix. These features were subsequently fed into a multi-class SVM for the classification task.

Choudhury et al.^49^ introduced a method for classifying skin cancer images, employing a multilayer decomposition approach based on textural and color features. Initially, images underwent decomposition into a piecewise base layer and detail layer using the weighted least squares (WLS) framework for edge-preserving decomposition. From the enhanced layer, GLCM and histogram of oriented gradients (HOG)^50^ served as textural feature descriptors, while the color histogram^51^ obtained from the base or smoothened layer was considered as the color feature descriptor. These feature values were passed as input into a multiclass SVM and extreme learning machine (ELM) for classification. The achieved accuracy was 94.18% with SVM and 90.5% with ELM, respectively. Bareiro et al.^52^ introduced an automated system utilizing a set of handcrafted features and an ML classifier for the detection of benign and malignant skin cancer from dermoscopic images. The proposed system employed various feature extraction techniques, including the Otsu algorithm^53^, asymmetry, border, color, and diameter (ABCD) rules^54^, inpainting techniques^55^, median filter^56^, and contrast limited adaptive histogram equalization (CLAHE)^57^. The Otsu algorithm played a crucial role in automated image segmentation, facilitating the separation of the region of interest (ROI) from the background, specifically aiding in isolating the lesion area for subsequent analysis. The ABCD rules align with established clinical assessment guidelines for melanoma, potentially improving accuracy. Inpainting techniques and median filter were responsible for removing unwanted artifacts and noise from the images, while CLAHE was used to enhance the contrast of the images. The classification model utilized an SVM as the classifier. The evaluation of this model on a self-procured dataset, consisting of 104 dermoscopic images, resulted in a classification accuracy of 90.63%.

**Fig. 5:**
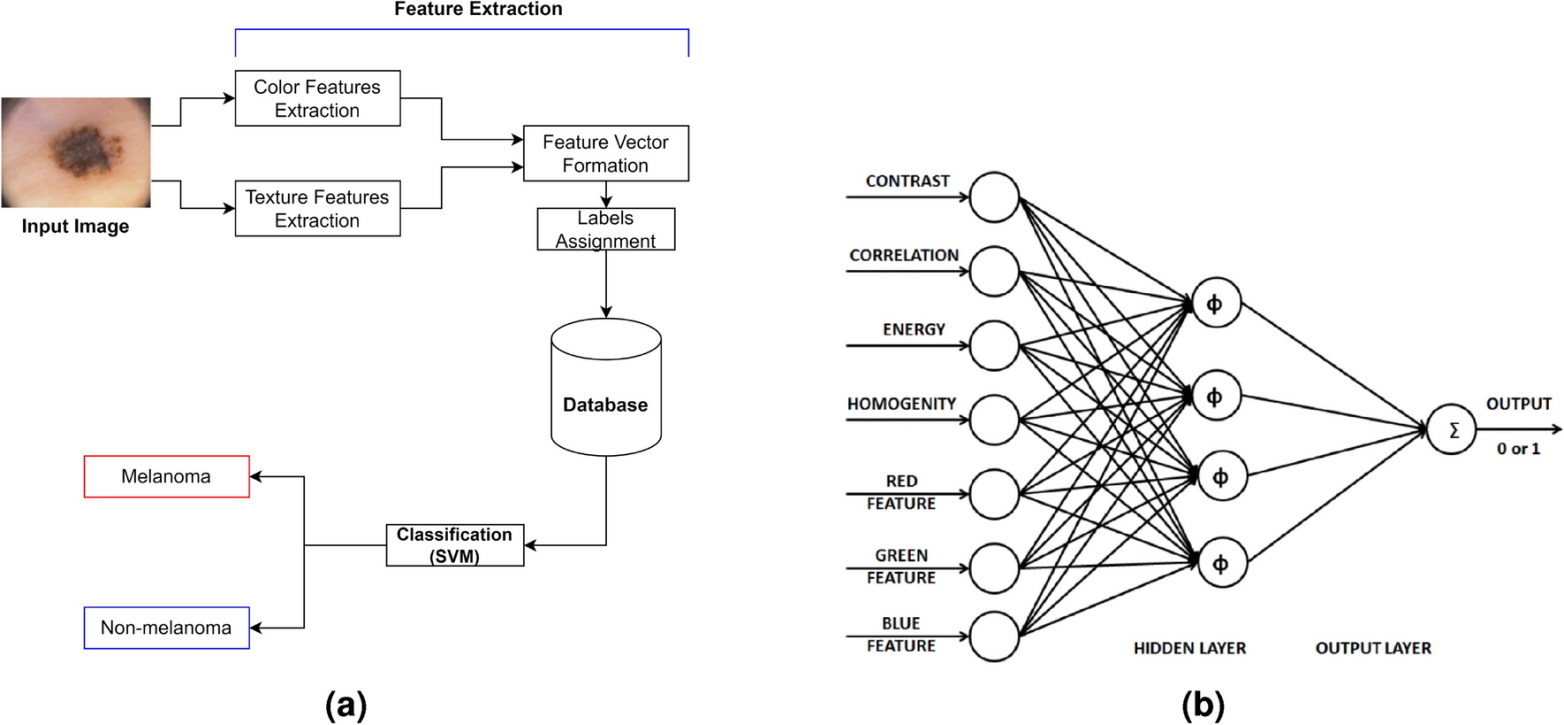
(**a**) Flowchart of the skin cancer classification system proposed by Waheed et al.^58^; (**b**) Structure of the hybrid GA-ANN classifier proposed by Aswin et al.^85^.

Waheed et al.^58^ proposed an effective ML model designed for the early diagnosis of skin cancer using dermoscopic images from patients. In this model, the feature extraction phase utilized the uniform HSV color space^59^ and the GLCM. For classification, an SVM was employed. The use of the HSV color space facilitated a focused analysis of color variations, crucial for identifying color-based characteristics associated with different skin lesions. GLCM was instrumental in understanding spatial relationships between pixel values, aiding in the extraction of texture features. The model underwent training and testing on 200 dermoscopic images from the PH2 dataset, achieving an impressive accuracy of 96% during experimentation and classification. The flowchart of this system is shown in Fig. 5a. Ozkan IA and Koklu^60^ presented an ML-based decision support system designed to assist doctors and radiologists. The feature extraction phase of this system utilized the ABCD rules-based technique, similar to Bareiro et al.’s work^52^. For classification, four different classifiers, namely ANN, SVM, k-NN, and DT, were employed. The system underwent evaluation on 200 dermoscopic images obtained from the PH2 dataset, achieving classification accuracies of 92.50%, 89.50%, 82%, and 90%, respectively, during experimentation.

Tan et al.^61^ proposed an automated ML system for skin cancer diagnosis using dermoscopic images. The model incorporated various feature extraction techniques, including gray-level run-length matrix (GLRLM) , ABCD rules, local binary patterns (LBP)^42^, and HOG, and used particle swarm optimization (PSO)^62^ for feature selection. The ABCD rules captured shape and color features, GLRLM focused on texture information, LBP captured local patterns, and HOG extracted gradient-based features. PSO optimized feature selection, enhancing the overall feature set. The integration of these diverse features aimed to provide a comprehensive representation of the underlying characteristics of skin lesions. These features were then combined with SVM and k-NN ensembles for classification. The model was evaluated on 1500 skin lesion images of patients taken from two datasets, PH2 and Dermofit, and produced classification accuracy of 97.79% and 97.54% under SVM and k-NN respectively.

Gautam et al.^63^ utilized LBP, uniform LBP^64^ and complete LBP (CLBP)^65^ as feature extraction methods. The features extracted from each of these methods were separately fed into DT, RF, SVM, and k-NN classifiers. The findings suggest that a combination of CLBP and RF yielded the best accuracy. Javaid et al.^66^ introduced a methodology that involved the integration of image processing and machine learning classifiers. The approach featured an innovative technique for contrast stretching of dermoscopic images, based on the mean values and standard deviation of pixels. Subsequently, the Otsu thresholding algorithm was employed to binarize the images. Then, features such as GLCM for texture identification, HOG for object identification, and color features were extracted from the images. Dimensionality reduction on the extracted features was carried out using principal component analysis (PCA)^67^. The feature vector underwent standardization and scaling. Prior to employing classifiers, a distinct wrapper method for feature selection was proposed. The effectiveness of the proposed approach was assessed on the ISIC 2016 dataset, achieving a maximum accuracy of 93.89% with the RF classifier.

**Table 3:** A comparative analysis of skin cancer classification methods using traditional ML classifiers.

Authors	Type	Dataset	Results
Jørgensen et al.^40^, 2008	akiec/bcc	78 OCT images	Acc-0.770
Zortea et al.^41^, 2010	Malignant/benign	217 images	Sen-0.730, Spe-0.730
Ballerini et al.^43^, 2012	akiec/bcc/bkl/nv/scc	960 images	Acc-0.740
Mhaske et al.^45^, 2013	Malignant/benign	104 images	Acc-0.800 to 0.900
Maurya et al.^47^, 2014	akiec/bcc/mel/scc	DermNet	Acc-0.814
Choudhury et al.^49^, 2015	akiec/bcc/mel/scc	DermNet	Acc-0.942
Bareiro et al.^52^, 2016	Malignant/benign	104 images	Acc-0.906
Waheed et al.^58^, 2017	mel/non-mel	PH2	Acc-0.960
Ozkan et al.^60^, 2017	Normal/abnormal/mel	PH2	Acc-0.925
Tan et al.^61^, 2019	Malignant/benign	PH2, Dermofit	Acc-0.978
Gautam et al.^63^, 2020	Malignant/benign	ISIC archive (947 images)	Acc-0.803
Javaid et al.^66^, 2021	mel/non-mel	ISIC 2016	Acc-0.939

**Observations:** Handcrafted features in skin cancer classification are typically categorized into color, shape, and texture features, each of which plays a crucial role in characterizing skin lesions. Color features, such as mean color values and color histograms, were employed by^43^ and^49^, respectively, to capture the distribution of colors in lesions. Shape features, which focus on geometrical aspects and lesion edges, were extracted using methods like the Otsu algorithm by^52,63^. Texture features, which analyze the spatial arrangement of pixel intensities, were explored through various techniques. For instance, GCM was used by^43^, GLCM was widely used by^47,49,58,63^, while the GLRLM and LBP were employed by^61^ and^61,63^, respectively. LBPH and HOG were used by^41^ and^49,61,63^, respectively. Additionally, wavelet transform techniques, which provide multi-resolution analysis, have been utilized by^45^. ML-based systems incorporating handcrafted features are easy to implement, more interpretable, computationally efficient and often require less data for training compared to deep learning methods. However, handcrafted features are manually designed based on prior knowledge, which might not fully capture the intricate patterns and representations present in skin lesions. The effectiveness of these techniques heavily depends on the quality of the manually engineered features. Designing relevant features requires domain expertise and might be challenging due to the variability in lesion appearances.

References^40,41,43,45,52^ use a self-procured dataset for testing, limiting their comparison with other studies. While^45,52,58,60^ yield high accuracies, their evaluation on smaller datasets raises concerns about their robustness for real-world scenarios. The absence of experimental results on larger datasets questions the generalizability of these models.^47,63^ uses a larger dataset to test their method. Nevertheless, their method fails to achieve high accuracy. In contrast, the improved accuracy over a relatively larger number of images demonstrated in^49,61,66^ indicates the effectiveness of the proposed schemes, surpassing other ML-based systems. This highlights their potential for robust skin cancer classification. However, it is important to note that computing features using a combination of multiple handcrafted feature extraction techniques like ABCD rules, GLCM, GLRLM, LBP, HOG etc. can lead to increased computational complexity. Therefore, the utilization of optimal feature selection techniques, as demonstrated in^61,66^, becomes essential.

#### Artificial neural network-based techniques

ANNs coupled with handcrafted features offer ease of implementation, interpretability, and computational efficiency. Skin cancer classification often involves complex patterns and non-linear relationships within imaging data. ANNs, with their inherent non-linearity, excel at automatically learning relevant features from raw pixel data. This is advantageous in skin cancer classification tasks where manual feature engineering techniques may not capture the diverse and subtle characteristics of lesions^68^. Additionally, ANNs have demonstrated strong generalization capabilities using large labelled datasets. In skin cancer classification, where diverse cases are encountered, ANNs can generalize well to new, unseen examples. A comprehensive comparison of skin cancer classification methods using ANNs is discussed in Table 4.

Ercal et al.^69^ investigated the efficacy of ANNs in analyzing tumor shape and relative tumor color to differentiate between benign and malignant skin lesions. The study involved the development and assessment of neural network models trained on color images of skin lesions to precisely classify them as malignant or benign, contributing to improved diagnostic accuracy in melanoma detection. Bayot et al.^70^ underscored the significance of identifying malignancy in individuals at risk of basal cell carcinoma through the integration of image processing techniques and ANNs. The image processing approach incorporated histogram equalization^71^ to enhance the contrast of the images. Lau et al.^72^ also employed histogram equalization to enhance the images, with the resulting enhanced grayscale image serving as the model input. They utilized the 2D wavelet decomposition technique to extract relevant cancer-related features from the images, avoiding dependence on clinical knowledge. These features were then passed as input into a backpropagation neural network comprising 3 layers, and an auto-associative neural network. The achieved accuracies were 89.90% and 80.80%, respectively. Mahmoud et al.^73^ conducted a study centred on automatically identifying melanoma through the utilization of wavelet^74^ and curvelet^75^ analyses. This led to the advancement of the exploration of sophisticated image analysis techniques combined with neural networks for more accurate and early identification of melanoma.

Jaleel et al.^76^ introduced an automated skin cancer diagnostic system utilizing an ANN based on backpropagation. The model they proposed utilized a 2D wavelet transform technique for feature extraction, allowing for the representation of both spatial and frequency information, enabling a more comprehensive analysis of texture and structural patterns in dermoscopy images. This system was designed to categorize all input images into two classes: cancerous and non-cancerous. Subsequently, Jaleel et al.^77^ also employed the GLCM technique for feature extraction and fed the extracted features into an ANN with backpropagation. Similar to the approach by Jaleel et al.^77^, Mabrouk et al.^78^ also utilized GLCM for extracting texture features. They extracted a total of 23 GLCM features, and, following Fisher’s scoring method^79^, 11 features were selected, which subsequently formed the input to an ANN. Masood et al.^80^ introduced an automated skin cancer diagnostic system based on ANNs. The study delved into the effectiveness of three ANN learning algorithms: Levenberg-Marquardt (LM)^81^, resilient backpropagation (RBP)^82^, and scaled conjugate gradient (SCG)^83^. The comparative analysis revealed that the LM algorithm achieved the highest specificity score at 95.10%. It was particularly efficient in classifying benign lesions. The LM algorithm is known for its fast convergence and it tends to perform well when dealing with small to medium-sized datasets, which is often the case in skin cancer classification tasks. Additionally, it was observed that increasing the number of epochs led to improved results with the SCG learning algorithm, achieving a sensitivity value of 92.60%.

Choudhari et al.^84^ proposed an ANN-based diagnostic system involving lesion isolation using a maximum entropy thresholding measure. Then they utilized GLCM to extract distinctive features from the segmented images. Subsequently, a feed-forward ANN classified the input images into either a malignant or benign stage of skin cancer, achieving an accuracy of 86.66%. Aswin et al.^85^ developed a novel skin cancer detection method that incorporated genetic algorithms (GA)^86^ and ANNs. Their model included hair removal as a preprocessing step, executed through the medical imaging software, DullRazor^87^. Additionally, the ROI was isolated using the Otsu thresholding method. Unique features of skin lesions were then extracted using the GLCM technique followed by optimal feature selection using GAs. Ultimately, the proposed model utilized an ANN to classify images into cancerous and non-cancerous categories and achieved an overall accuracy score of 92.30%. The structure of the hybrid GA-ANN classifier is shown in Fig. 5b. Xie et al.^88^ introduced a skin lesion classification system designed to categorize lesions primarily into malignant and benign classes. The system operated through three key stages. Initially, a self-generating neural network was employed for lesion extraction from images. Following this features related to tumor border, color, and texture details were extracted, totalling 57 features, with 7 novel features specifically focused on lesion borders. PCA was then applied for dimensionality reduction to identify the most optimal feature set. In the final stage, classification was carried out using an ensemble neural network model that combined backward propagation neural networks and fuzzy neural networks. The model’s classification performance was compared with other classifiers such as SVM, k-NN, RF, and AdaBoost. The proposed model exhibited approximately 7.50% higher sensitivity compared to alternative classifiers and achieved an impressive accuracy rate of 91.11%.

In the research conducted by Kanimozhi et al.^89^, the ABCD rules were employed for extracting features from lesion images. Their study focused on leveraging ANN with suitable backpropagation algorithms to assist in the accurate and automated detection of melanoma, contributing to improving diagnostic capabilities specifically for this type of skin cancer. The paper by Cueva et al.^90^ introduced a mole classification system designed for the early diagnosis of melanoma skin cancer. This system extracted features based on the ABCD rules of lesions, focusing on asymmetry, borders, color, and diameter of moles. Asymmetry was determined using the Mumford-Shah algorithm^91^, while the Harris–Stephens algorithm^92^ extracted mole borders. Moles with colors other than black, cinnamon, or brown were considered potential indicators of melanoma. Additionally, melanoma moles typically have a diameter exceeding 6 mm, which serves as a threshold for their detection. The proposed system utilized a feed-forward backpropagation ANN to classify moles into common mole, uncommon mole, or melanoma mole categories, achieving an accuracy of 97.51%.

**Table 4:** A comparative analysis of skin cancer classification methods using ANNs.

Authors	Type	Dataset	Results
Ercal et al.^69^, 1994	Malignant/benign	326 images	Acc-0.800
Bayot et al.^70^, 2007	bcc/non-bcc	180 images	Acc-0.933
Lau et al.^72^, 2009	Malignant/benign	448 images	Acc-0.899
Mahmoud et al.^73^, 2011	Malignant/benign	448 images	Acc-0.704
Jaleel et al.^76^, 2012	Malignant/benign	31 images	Acc-0.867
Jaleel et al.^77^, 2013	Malignant/benign	50 images	Acc-0.880
Mabrouk et al.^78^, 2013	mel/nv	140 images	Acc-0.910
Masood et al.^80^, 2014	mel/non-mel	135 images	Acc-0.951
Choudhari et al.^84^, 2014	mel/non-mel	90 images	Acc-0.867
Aswin et al.^85^, 2014	Malignant/benign	30 images	Acc-0.923
Xie et al.^88^, 2016	Malignant/benign	Caucasian race and Xanthous race	Acc-0.911, Sen-0.950, Spe-0.938
Kanimozhi et al.^89^, 2016	mel/non-mel	31 images	Acc-0.969
Cueva et al.^90^, 2017	Common mole/uncommon mole/mel	200 images	Acc-0.975

**Observations:** Like ML classifier-based techniques, ANNs also utilize handcrafted features. GLCM was employed by^77,78,84,85^. Wavelet and curvelet techniques were used by^72,76^ and^73^, respectively. The Otsu algorithm was employed by^85^. While the combination of ANNs with handcrafted features has proven effective in capturing non-linear relationships within image features, these systems share many limitations with ML-based approaches. The manual choice and design of features can be a subjective process, varying among researchers. This subjectivity introduces bias and inconsistency in feature extraction, potentially reducing classification accuracy in real-world scenarios.

References^69,70,72,73,76–78,80,84,85,88–90^ all utilize proprietary datasets for testing their methods, limiting comparisons with other methods. While^69,70,73,80^ produce fairly decent results, they do not emphasize the creation of an effective set of image features for utilization by their ANN models. On the other hand, Refs.^72,76,77,84,89,90^ incorporate sophisticated image processing techniques to capture features but do not focus on optimal feature selection, potentially leading to computational inefficiency due to increased features. In contrast, Refs.^85,88^ employ GA and PCA-based techniques for feature selection and feature reduction, respectively, resulting in improved results. Mabrouk et al.^78^ also emphasizes the importance of optimal feature selection through the application of Fisher’s scoring technique. However, it is important to note that handcrafted features struggle to capture intricate and complex patterns present in skin lesions, especially when dealing with subtle or non-obvious visual cues. This limitation can impact the model’s capacity to accurately distinguish between malignant and benign lesions, and it becomes even more pronounced in the context of multi-class classification of skin cancer, despite careful feature selection.

#### Kohonen network-based techniques

Kohonen networks^93^ offer an alternative to ANNs and traditional ML classifiers when incorporating handcrafted features for classification tasks. Renowned for their ability to preserve the topology of input data, Kohonen networks prove advantageous in skin cancer classification by maintaining spatial relationships and structures within the feature space, potentially capturing essential contextual information. In contrast to ANNs and ML classifiers, Kohonen networks inherently perform dimensionality reduction as they map high-dimensional input data to a lower-dimensional grid. Moreover, these networks naturally cluster similar patterns together on the map, providing an intuitive means to visualize the distribution of different lesion types. This clustering feature aids in identifying distinct groups and patterns within the dataset. A comprehensive comparison of skin cancer classification methods utilizing Kohonen networks is discussed in Table 5.

In their research, Lenhardt et al.^94^ presented a skin cancer detection system centred around Kohonen networks. The study involved the utilization of synchronous fluorescence spectra from melanoma, nevus, and normal skin samples for training the network. To capture the fluorescence spectra of these samples, obtained from human patients immediately post-surgery, a fluorescence spectrophotometer was employed. The dimensionality of the measured spectra was reduced through PCA. Following this, both Kohonen networks and ANNs underwent training using this dataset. Kohonen networks demonstrated superior performance compared to ANNs, exhibiting lower classification errors. Mengistu et al.^95^ introduced a skin cancer detection system that integrated Kohonen networks and radial basis function (RBF) neural networks. The input for this system consisted of color information, along with features derived from GLCM analysis and morphological characteristics extracted from lesion images. The performance of the proposed system was then compared to other classifiers, including k-NN, ANN, and Naive Bayes classifier. The results showcased that the amalgamation of the Kohonen network and RBF neural network achieved an impressive accuracy of 93.15% and outperformed the other classifiers.

In their work, Sajid et al.^96^ introduced a skin cancer diagnostic system leveraging Kohonen networks. The authors implemented a median filter to efficiently eliminate noise from the images. For image segmentation, a region growing and merging algorithm^97^ was employed on the filtered images. The system utilized a combination of textual and statistical features for classification, with statistical features extracted from lesion images and textual features extracted from the curvelet domain. The primary goal was to categorize input images into malignant and benign classes. The proposed model demonstrated an exceptional accuracy of 98.30%. In evaluating the system’s performance, it was compared with other classifiers, including SVM, a backpropagation neural network, and a 3-layered neural network. The results indicated that SVM achieved an accuracy of 91.10%, the neural network with backpropagation reached 90.40% accuracy, and the 3-layered neural network attained 90.50% accuracy. Notably, these accuracies were considerably lower than the accuracy achieved by the proposed system.

**Table 5:** A comparative analysis of skin cancer classification methods using Kohonen networks.

Authors	Type	Dataset	Results
Lenhardt et al.^94^, 2013	Normal/mel/nv	50 images	Acc-0.960
Mengistu et al.^95^, 2015	bcc/mel/scc	DermQuest, DermNet	Acc-0.932
Sajid et al.^96^, 2018	Malignant/benign	500 images	Acc-0.983

**Observations:** While the ability of Kohonen networks to preserve intricate spatial relationships and perform feature reduction is advantageous for skin cancer classification, they also come with certain drawbacks. Their reliance on feature engineering and their inability to capture hierarchical relationships stand out as primary limitations^98^. Besides, unlike ANNs, Kohonen networks lack inherent support for end-to-end learning, limiting their adaptability to more complex data relationships.

References^94,96^ incorporate simplistic feature engineering methods for feature extraction and evaluate their models on self-procured datasets, limiting fair comparisons with other research. On the other hand, Mengistu and Alemayehu^95^ demonstrates high accuracy and superior performance of Kohonen networks over ANNs and traditional ML classifiers. Nevertheless, with the rise of deep CNNs, Kohonen networks have gradually lost their relevance.

### Deep learning-based techniques

**Fig. 6:**
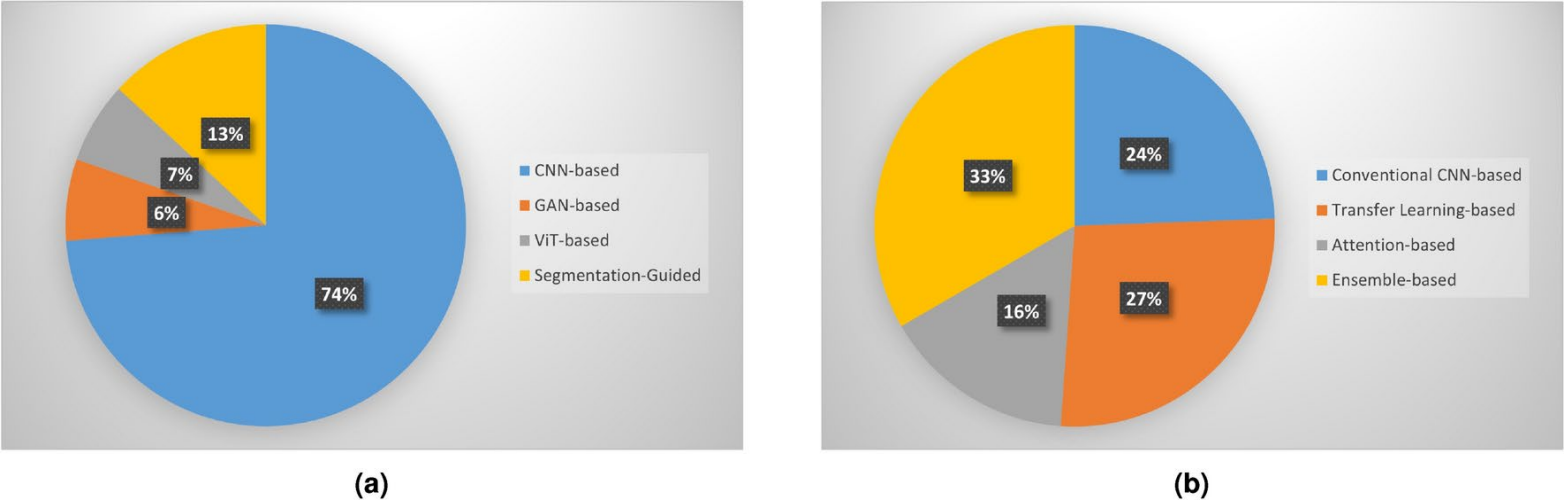
(**a**) Pie chart illustrating the percentage distribution of papers based on different DL models. (**b**) Pie chart illustrating the percentage distribution of papers based on various CNN-based techniques.

The emergence of DL, a specialized subset of ML, has yielded rapid growth in the fields of pattern learning, image classification and recognition. DL models are trained on input data and not programmed explicitly. After the training phase, these models act as experts in the domain in which they were trained. Deep neural networks play an important role in the classification of skin cancer. In this section, we discuss various types of DL techniques that have been trained to classify images and distinguish between different types of skin cancer. Figure 6a presents a pie chart illustrating the proportions of research papers based on various DL models. Notably, papers utilizing CNNs constitute over 70% of the surveyed literature.

#### Convolutional neural network-based techniques

In the field of medical imaging, CNNs have demonstrated exceptional performance in tasks involving detection, segmentation, and classification. For an in-depth understanding of CNNs’ automated feature extraction abilities, readers may refer to key studies in^99^. CNNs play a significant role in skin cancer classification due to their ability to automatically learn intricate patterns and features from images. Unlike traditional methods that rely on handcrafted features, these neural networks are adept at capturing hierarchical features within the numerous lesion images, recognizing patterns at various levels of abstraction^100^. Initially, CNNs detect simple features like edges and textures, and as the network deepens, they capture more complex structures, such as irregularities in symmetry, borders, textures, and other visual cues crucial for distinguishing between different types of skin lesions. By stacking convolutional and pooling layers, CNNs gradually refine their feature detection, allowing them to identify subtle differences in lesion appearance that might not be apparent to the naked eye. This ability enables more accurate diagnosis and can significantly assist dermatologists in identifying early signs of skin cancer. In this section, we present a comprehensive discussion of the several CNN-based methods extensively used for skin cancer classification. Figure 6b presents a pie chart illustrating the proportions of papers using various CNN-based techniques.

##### Conventional CNN-based techniques

This section outlines the various custom CNN architectures devised by researchers for skin cancer classification. Nasr-Esfahani et al.^101^ developed a CNN with the aim of enhancing the accuracy and efficiency of detecting melanoma through automated analysis of clinical images. The CNN consisted of 2 convolutional layers to capture patterns from the images, 2 max-pooling layers to reduce the size of the feature maps, a fully connected layer and a final output layer containing 2 neurons, representing the categories of malignant and benign. Sabouri et al.’s work^102^ involved the development and training of CNNs to accurately identify and outline lesion borders within medical imaging data. The main objective of this work was to improve the precision and automation of lesion border detection, contributing to enhanced medical image analysis techniques for diagnosing and understanding various medical conditions. Ali et al.^103^ developed LightNet, a CNN with fewer parameters and suitable for mobile applications. The study utilized a conventional CNN architecture featuring 5 convolutional layers, 3 max-pooling layers, and 2 fully connected layers. To limit the parameters, they maintained a moderate number of filters in the convolutional layers. Batch normalization was applied after each convolutional layer to expedite convergence and impose regularization. Additionally, dropout was implemented in the fully connected layers to mitigate overfitting.

Esteva et al.^104^ employed a combination of convolutional layers, pooling layers, Inception modules, and residual connections allowing the network to learn powerful features from skin lesion images and to achieve high accuracy in skin cancer classification. The Inception modules helped to combine convolutional filters in parallel for multi-scale feature extraction. Also, they used global average pooling instead of fully connected layers to reduce the network’s complexity and avoid overfitting. Ayan et al.^105^ designed a CNN comprising 11 layers and emphasized the significance of data augmentation for constructing a robust skin cancer classifier. They applied various image augmentation techniques, including random transformations, rotations at different angles, shifting, zooming, and flipping. The classifier achieved an accuracy of 78% on the original dataset and an accuracy of 81% on the augmented dataset. Mandache et al.^106^ developed a CNN with a series of convolutional layers with varying filter sizes and non-linear activation functions to extract relevant features from 40 full field OCT (FF-OCT) images. These features capture various aspects of the basal cell carcinoma morphology such as loss of normal skin layering, presence of cystic spaces and retraction of the epidermal-dermal junction.

Along with the conventional convolutional and pooling layers, Namozov et al.^107^ utilized a parameterized activation function called the adaptive piecewise linear unit (APLU). APLU consists of adjustable parameters which allows the model to learn more complex and nuanced decision boundaries, potentially leading to improved feature discrimination and classification accuracy. In a study conducted by Ahmed et al.^108^, a standard CNN featuring multiple convolutional and pooling layers was utilized to classify lesion images sourced from the ISIC archive. The researchers also conducted experiments with Naive Bayes, SVM, k-NN classifiers, with CNNs demonstrating superior performance. Mridha et al.^109^ introduced a customized CNN architecture comprising two blocks for the feature extraction phase. In block 1, there were 2 convolutional layers with a kernel size of 3, accompanied by a pooling layer with a stride of 1, and a dropout layer. Block 2 included two convolutional layers with a kernel size of 3, a pooling layer with a stride of 2, and a dropout layer. The output from block 2 was flattened and subsequently passed through a final dense layer.

**Fig. 7:**
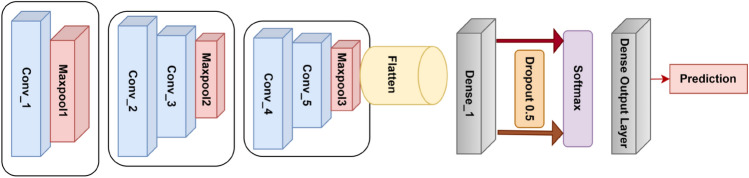
Architecture of the SkinNet-8 model proposed by Fahad et al.^110^.

SkinNet-8, designed by Fahad et al.^110^, is a relatively simple yet computationally efficient CNN with 10 layers, including 5 convolutional layers, 3 pooling layers, and 2 dense layers. All these layers have been organized into 3 blocks. The model begins with an input image of fixed size, processed through the first block, which consists of a single convolutional layer followed by a max-pooling layer. The output from the first block feeds into block two and block three, each composed of two convolutional layers and a max-pooling layer. The resulting feature maps from the last block are flattened into a 1D vector, which is connected to dense layers. Finally, a softmax activation function is utilized to perform binary classification. It achieved a remarkable test accuracy of 98.81% on the imbalanced ISIC 2020 dataset. Figure 7 shows the architecture of SkinNet-8. Rastegar et al.^111^ proposed a deep CNN with 69 layers, aiming to extract detailed and discriminative features from skin lesion images. The network consists of multiple convolutional layers with different filter sizes, $3 \times 3$, $5 \times 5$, $7 \times 7$, and depths. The network also contains residual layers, Inception modules and pooling layers accompanied by a final fully connected classification layer. A comprehensive comparison of skin cancer classification methods using conventional CNNs is discussed in Table 6.

**Table 6:** A comparative analysis of skin cancer classification methods using conventional CNN-based techniques.

Authors	Type	Dataset	Results
Nasr-Esfahani et al.^101^, 2016	Malignant/benign	170 images	Acc-0.810, Sen-0.810, Spe-0.800
Sabouri et al.^102^, 2016	mel/non-mel	1730 images	Acc-0.867
Ali et al.^103^, 2017	Malignant/benign	ISIC 2016	Acc-0.816, Sen-0.149, Spe-0.980
Esteva et al.^104^, 2017	Malignant/benign, bkl/benign	ISIC archive	Acc-0.721
Ayan et al.^105^, 2018	malignant/benign	ISIC dataset (1000 images)	Acc-0.810
Mandache et al.^106^, 2018	bcc/benign	40 images	Acc-0.959, Sen-0.952, Spe-0.965
Namozov et al.^107^, 2018	akiec/bcc/df/mel/nv/vasc	ISIC 2018	Acc-0.959, Pre-0.955, Sen-0.955, Spe-0.955, AUC-0.98
Ahmed et al.^108^, 2023	Malignant/benign	ISIC archive (2635 images)	AUC-0.995
Mridha et al.^109^, 2023	akiec/bcc/df/mel/nv/vasc	HAM10000	Acc-0.820
Fahad et al.^110^, 2023	Malignant/benign	ISIC 2020	Acc-0.988, Pre-0.988, Sen-0.988, Spe-0.988, F1 score-0.988
Rastegar et al.^111^, 2023	Malignant/benign	ISIC 2016, ISIC 2017, PH2	ISIC 2016: Acc-0.952, ISIC 2017: Acc-0.994, PH2: Acc-0.972

**Observations:** While conventional CNNs have shown promise in skin cancer classification, they often operate with fixed-size convolutional filters, making it challenging to capture long-range dependencies or understand the global structure of large images. Additionally, the pooling layers employed in CNNs decrease the spatial resolution of feature maps, resulting in information loss. This decrease in spatial resolution might discard crucial fine-grained details necessary for precise skin cancer classification.

References^101,102,106^ make use of proprietary datasets for testing their CNN architectures, limiting comparative analysis with other models. While Ali and Al-Marzouqi^103^ might prove useful for mobile applications, it renders low accuracy due to less number of filters in the convolutional layers. Although Ayan and Ünver^105^ does not achieve high accuracy, attributed to the simplistic CNN architecture employed, it still demonstrates satisfactory results, emphasizing the crucial role of data augmentation in this domain. The integration of Inception modules into CNNs, as demonstrated by^104^, stands as a crucial advancement, adopted in subsequent research. Namozov and Im Cho^107^ yields good results on the challenging ISIC 2018 dataset. However, they do not consider the underrepresented class of benign keratosis. References^108,110,111^ produce impressive binary classification results through relatively simple architectures. Moreover, the study conducted in^108^ indicates the superior performance of DL-based CNN models over traditional ML classifiers. However, they do not evaluate their methods for multi-class classification scenarios. With a simple CNN architecture, Mridha et al.^109^ yields low accuracy for multi-class classification. This underscores the limitation of simple conventional CNNs in effectively classifying diverse categories of skin lesions.

##### Transfer learning-based techniques

Transfer learning (TL) plays a pivotal role in skin cancer classification by leveraging knowledge learned from pre-trained models on large, diverse datasets to improve the performance of models trained on smaller skin cancer datasets. This approach is particularly beneficial due to the limited availability of skin cancer-related data. TL also leads to faster training times^112^. However, it is important to recognize that pre-trained models are typically trained on datasets like ImageNet, which consists of everyday objects, scenes, and animals. In contrast, medical images, especially dermoscopic skin lesion images, are highly specialized and characterized by unique patterns, textures, and color variations that are tied to biological factors. This difference leads to a domain shift between natural and medical images, making it challenging for pre-trained models to generalize effectively. If this domain shift is not addressed, models trained on natural images may fail to capture essential diagnostic features in medical images, resulting in poor classification performance.

To mitigate this, fine-tuning the pre-trained models on skin lesion datasets is an effective strategy. By gradually updating the model’s weights, the model can adapt to the new domain while retaining useful knowledge from the original one. A common technique is to freeze the lower layers of the pre-trained model, responsible for capturing general features like edges and textures, and only fine-tune the higher layers that learn task-specific features. This approach helps prevent overfitting on the small medical dataset while enabling the model to better align with the new domain. A comparative analysis of various skin cancer classification methods based on TL is presented in Table 7.

A pre-trained deep CNN architecture, VGG16 with the last 3 fine-tuned layers and 5 convolutional blocks was proposed by Kalouche et al.^113^. For fine-tuning, they employed a stochastic gradient descent (SGD) optimizer with a low learning rate. This model built on VGG16 produced 78% accuracy for melanoma classification. De Vries and Ramachandram^114^ introduced a multi-scale CNN utilizing the InceptionV3 architecture. They fine-tuned the pre-trained InceptionV3 model on two distinct resolution scales of input lesion images: a coarse scale and a finer scale. The multi-scale network is established by initially processing both the low-resolution image and the high-resolution image using the same InceptionV3 feature extractor. The resulting feature vectors from each image are combined to form a singular 4096 element vector. This combined vector then undergoes processing through a fully connected layer. Ultimately, a three-way softmax function is applied to generate probability predictions for the three classes: melanoma, seborrheic keratosis, and nevus.

Like Kalouche et al.’s approach^113^, Lopez et al.^115^ proposed a deep CNN built on the VGG16 architecture. This pre-trained model was then fine-tuned by replacing the last 2 fully connected layers with new layers specific to the binary classification task. Additionally, they replaced the activation function in the modified layer from softmax to sigmoidal. Mendes and da Silva^116^ proposed a deep CNN architecture based on pre-trained ResNet152 to classify 12 different kinds of skin lesions. Initially, the proposed model was trained on 3797 lesion images collected from the MED-NODE, Dermofit, and AtlasDerm datasets. Later, 29 times augmentation was applied depending on lighting positions and scale transformations. Hosny et al.^117^ utilized the pre-trained AlexNet architecture for feature extraction while developing their classification model. Here, the first few layers of AlexNet are kept frozen (not further trained), while the last layers are replaced with a new softmax layer. This new layer combines the extracted features to classify melanoma, common nevus and atypical nevus lesions.

Rezvantalab et al.^118^ employed 4 deep CNNs, namely, InceptionV3, InceptionResNetV2, ResNet152, and DenseNet201. Each network underwent fine-tuning across all layers, with the top layers replaced by a global average pooling layer and a softmax layer. DenseNet201 demonstrated superior performance with an AUC score of 0.979. The study also compared these networks’ performance with highly trained dermatologists, revealing that the networks outperformed dermatologists by at least 11%. Emara et al.^119^ employed the InceptionV4 backbone and introduced modifications by incorporating feature reuse through a residual connection. This connection played a crucial role in merging features extracted from earlier layers with those from high-level layers, contributing to an enhancement in the classification performance of the model on the challenging ISIC 2018 dataset. Gulati et al.^120^ explored two ways of using pre-trained models. Similar to Hosny et al.’s work^117^, they used fine-tuned AlexNet. They also used VGG16 as a feature extractor. Here, instead of fine-tuning, the features extracted by the layers of VGG16 are fed into a new fully connected layer trained specifically for melanoma classification. The modified VGG16 network outperformed AlexNet and achieved an accuracy of 97.50% on the PH2 dataset.

Le et al.^121^ utilized the ResNet50 backbone with additional modifications for the classification of 7 types of skin cancer. Their adaptations included the use of global average pooling instead of simple average pooling and the introduction of a dropout layer between the last 2 fully connected layers. Furthermore, they used a combination of weighted loss and focal loss to optimize their model. Sagar et al.^122^ employed several pre-trained models for the binary classification of melanoma. They performed experiments using InceptionV3, InceptionResNetV2, ResNet50, MobileNet and DenseNet169, out of which ResNet50 emerged with superior performance. Shen et al.^123^ leveraged a low cost and high performance data augmentation strategy along with TL for automatic skin cancer screening in rural communities. Their network, built on EfficientNetB7 architecture, achieved a multi-class classification accuracy of 85.30% on the HAM10000 dataset. Naeem et al.^124^ proposed an architecture based on the VGG16 model, enhancing its depth by adding two additional convolutional blocks. This modification was aimed at enabling the network to learn fine-grained features more effectively, thereby improving its capacity for detailed feature extraction for skin cancer classification.

**Table 7:** A comparative analysis of skin cancer classification methods using TL.

Authors	Type	Dataset	Results
Kalouche et al.^113^, 2016	Malignant/benign	ISIC database (1280 images)	Acc-0.780
De Vries et al.^114^, 2017	bkl/mel/nv	ISIC 2017	Acc-0.903, AUC-0.943
Lopez et al.^115^, 2017	Malignant/benign	ISIC 2016	Acc-0.813, Sen-0.786, Spe-0.840
Mendes et al.^116^, 2018	bcc/mel	MED-NODE, Dermofit	AUC (mel: 0.960, bcc: 0.910)
Hosny et al.^117^, 2018	mel/common nv/atypical nv	PH2	Acc-0.986, Pre-0.977, Sen-0.983, Spe-0.989
Rezvantalab et al.^118^, 2018	akiec/bcc/bkl/df/mel/ nv/atypical nv/vasc	HAM10000	Pre-0.890, AUC-0.979
Emara et al.^119^, 2019	akiec/bcc/bkl/df/mel/ nv/vasc	ISIC 2018	Acc-0.947, Sen-0.717, Spe-0.958, AUC-0.838
Gulati et al.^120^, 2019	mel/non-mel	PH2	Acc-0.975, Sen-1.000, Spe-0.969
Le et al.^121^, 2020	akiec/bcc/bkl/df/mel/ nv/vasc	HAM10000	Acc-0.900, Pre-0.810, Sen-0.800, F1 score-0.800
Sagar et al.^122^, 2020	mel/non-mel	ISIC database (3600 images)	Acc-0.935, Pre-0.940, Sen-0.770, F1 score-0.850
Shen et al.^123^, 2022	akiec/bcc/bkl/df/mel/ nv/vasc	HAM10000	Acc-0.853
Naeem et al.^124^, 2022	bcc/bkl/mel/nv	ISIC 2019	Acc-0.970, Pre-0.922, Sen-0.922, F1 score-0.922

**Observations:** While TL significantly contributes to skin cancer classification by harnessing the knowledge acquired from pre-trained models, sometimes, these pre-trained models might have been trained on datasets that do not align perfectly with the target task or have different classes. In such cases, pre-trained models might not adapt well to these differences and the relevance of the pre-trained features to the skin cancer classification task might be limited. Therefore, it becomes crucial to fine-tune the model appropriately. Inadequate fine-tuning choices could lead to a model that struggles to generalize effectively to the target dataset.

While emphasizing the significance of data augmentation, Mendes and da Silva^116^ does not assess their network’s performance on larger datasets, giving rise to concerns regarding its generalizability. Although Refs.^117,120^ achieve impressive results on the small PH2 dataset, like^116^, their networks are not tested on larger datasets, restricting broader evaluation. References^113,122,124^ demonstrate promising outcomes, but they evaluate their models on a subset of images rather than the entire dataset, making direct comparisons challenging. Although Refs.^113,115^ use the same network, Lopez et al.^115^ yields enhanced results over Kalouche et al.^113^ demonstrating the importance of suitable fine-tuning. The study conducted in^118^ demonstrates that TL-based networks surpassed dermatologists in achieving precise classification, thereby underscoring the significance of incorporating such models in a clinical setting. While Emara et al.^119^ presents a new perspective by introducing a modified Inception architecture along with residual connections, it is noteworthy that the sensitivity score of their model is relatively low. This poses a significant limitation, as misclassifying an individual with cancer as not having the condition carries a higher risk than the opposite scenario. Le et al.^121^ introduces a hybrid loss approach to tackle class imbalance. However, like Emara et al.^119^, the sensitivity score of their model is also observed to be low. DeVries and Ramachandram^114^ introduces an innovative multi-scale network that not only delivers impressive outcomes but also opens new pathways for models using feature fusion. The underwhelming multi-class classification outcomes observed on the challenging HAM10000 dataset in^123^ underscore the necessity for more effective strategies beyond vanilla TL-based approaches.

##### Attention-based techniques

Incorporating attention mechanisms within CNNs for skin cancer classification enhances models’ ability to concentrate on crucial features within the skin lesions by assigning weights to the feature maps according to their relevance to the lesions. This integration also helps suppress image artifacts, like portions of uninfected skin, hair, and veins, contributing to more accurate and precise diagnostic outcomes. A comparative analysis of various skin cancer classification methods based on attention mechanisms is presented in Table 8.

Zhang et al.^125^ proposed an attention residual learning CNN (ARL-CNN) model for the classification of skin lesions. This model comprised multiple ARL blocks, a global average pooling layer, and a classification layer. Each ARL block employed both residual learning and a unique attention learning mechanism to improve its capacity for capturing discriminative representations. The attention learning mechanism, rather than introducing extra learnable layers, aimed to leverage the inherent self-attention ability of deep CNNs. Specifically, it utilized feature maps learned by a higher layer to generate the attention map for a lower layer. Wu et al.^126^ introduced the ARDT-DenseNet, a densely connected convolutional network with attention and residual learning, for skin lesion classification. The ARDT block comprised dense blocks, transition blocks, and attention and residual modules. In comparison to a residual network with an equivalent number of convolutional layers, the parameter size of the proposed densely connected network was halved. The enhanced densely connected network incorporated an attention mechanism and residual learning after each dense block and transition block, providing additional functionality without introducing extra parameters.

Xue et al.^127^ introduced a novel network designed to differentiate between visually similar skin lesions, a challenging task for conventional neural networks. They utilized ResNet50 as the backbone network for extracting features from dermoscopic images. In addition to this, they developed a novel distinct region proposal module (DRPM), which is enhanced by the sequential computation of channel and spatial attention mechanisms. These attention mechanisms are crucial for focusing on critical areas within the lesions, allowing the model to identify and extract features from distinct regions that are particularly indicative of specific lesion types. Features extracted from these regions are then combined with those previously derived from the original dermoscopic images. This concatenated feature set forms the comprehensive input for the final classification task, aiming to accurately categorize skin lesions based on their subtle differences.

Ding et al.^128^ proposed the Deep Attention Branch Network (DABN) model, incorporating attention branches to enhance traditional deep CNNs. In the training stage, the attention branch was crafted to acquire the class activation maps, subsequently serving as attention maps directing the network’s focus to discriminative regions in skin lesions. DABN demonstrated applicability across diverse deep CNN structures and underwent end-to-end training. The DABN model incorporated 2 attention branches into the baseline architecture, which consisted of 4 dense blocks, 3 transition layers, and a classification layer. The dense block utilized the outputs of all preceding layers as input for each layer, promoting feature reuse and including multiple $1 \times 1$ and $3 \times 3$ convolutional layers. The transition layer incorporated a $1 \times 1$ convolutional layer and $2 \times 2$ average pooling to reduce the channel and size of the feature map. Finally, the classification layer employed global average pooling and 2 fully connected layers to generate the probability score for each category.

**Fig. 8:**
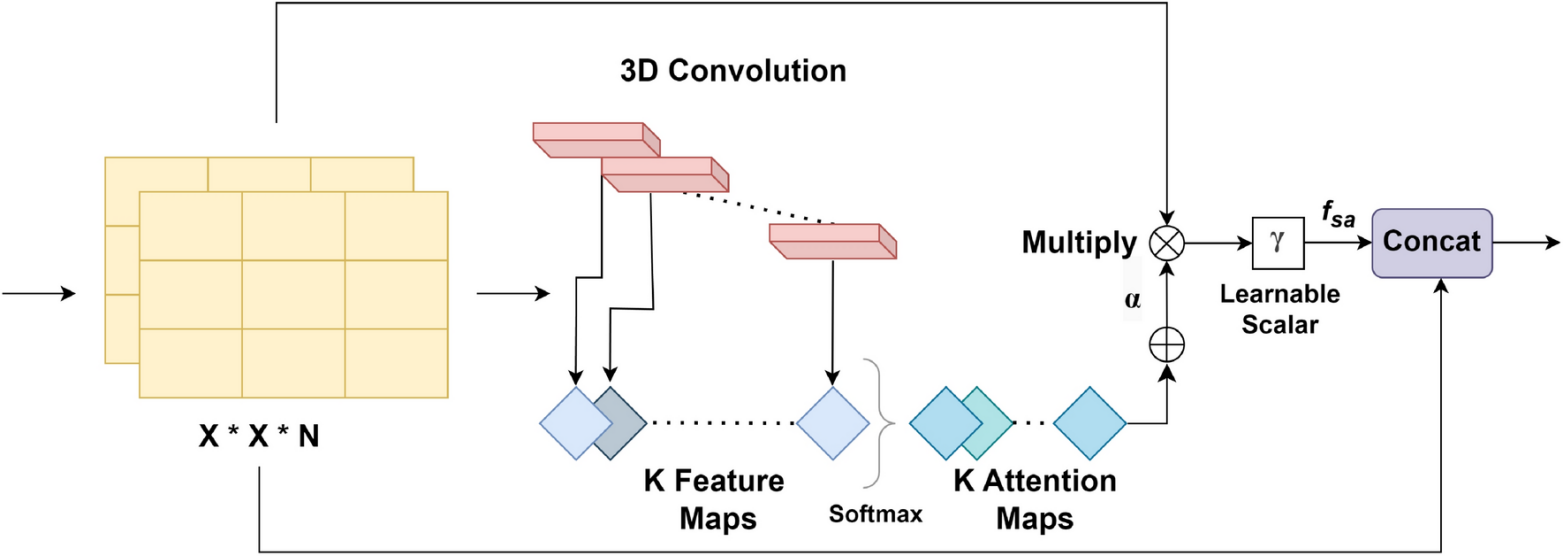
Overview of the soft attention unit proposed by Datta et al.^130^.

Following Xu et al.’s work^129^, Datta et al.^130^ proposed a skin cancer classification model using InceptionResNetV2 as backbone, aided with a soft attention unit. Here, the soft attention unit consists of two phases, a bilinear attention layer and a step to compute the weighted feature maps. Based on Eq. (1), the weighted feature maps are calculated by passing the feature tensor $t \in {\mathbb {R}}^{h\times w\times d}$ to a 3D convolution layer with weights $W_k \in {\mathbb {R}}^{h\times w\times d \times K}$, where *K* represents the number of 3D weights. Following this, a softmax function is applied to normalize each of the *K* attention maps. These normalized maps are then aggregated to create a composite attention map, which acts as a weighting function denoted as $\alpha$. This $\alpha$ value is used to scale the input tensor *t*, further adjusted by a trainable scalar $\gamma$. Ultimately, the scaled attentive features $f_{sa}$ are combined with the input tensor *t*. Figure 8 depicts the overview of this soft attention unit.

1 $$\begin{aligned} f_{s a}=\gamma t \left( \left( \sum _{k=1}^{K}softmax(W_k*t)\right) \right) \end{aligned}$$

Similar to Datta et al.’s work^130^, Alhudhaif et al.^131^ proposed an attention module, where they analyzed the feature maps and assigned weights based on their relevance to the lesion, highlighting important areas for further processing. They built their classification model by first employing two convolutional layers to extract basic features from the input images. Then, they added their attention block followed by four more convolutional layers. Finally, they used a multi-class prediction layer to obtain the output probabilities. They were able to achieve an impressive accuracy of 95.90% on the challenging HAM10000 dataset.

Roy et al.^132^ used the wavelet transform technique, a soft attention module, and their novel Symmetry-aware Feature Attention (SaFA) module for skin cancer classification. The SaFA module was designed to extract symmetry-related information from the lesions and detect semantic variations. This module consists of two key components: the Feature Difference-aware Block (FDaB) and the Symmetry-aware Block (SaB). The FDaB processes an input feature map with dimensions $H \times W \times C$ and reduces it to $H \times W \times 1$ using three separable convolution layers. The resulting feature map is reshaped into two feature maps of dimensions $H \times W$ and $W \times H$ respectively, which are fed into two long short-term memory (LSTM) layers to capture semantic changes across spatial dimensions $H$ and $W$. The outputs from these LSTM layers are reshaped back to $H \times W \times 1$ and concatenated to produce $F_{LSTM}$. The SaB takes this $F_{LSTM}$ as its input to generate $F_{symmetry}$, by calculating pixel-wise feature similarity between $F_{LSTM}$ and its transpose. This represents the symmetry-aware features. They first used a gradient-based fusion technique to fuse the features extracted by wavelet transform and soft attention and then concatenated it with the symmetry-aware features.

**Table 8:** A comparative analysis of skin cancer classification methods using attention-based CNN models.

Authors	Type	Dataset	Results
Zhang et al.^125^, 2019	bkl/mel/nv	ISIC 2017	Sen-0.770, AUC-0.920
Wu et al.^126^, 2020	Malignant/benign	ISIC 2016, ISIC 2017	ISIC 2016 (Acc-0.857, AUC-0.837), ISIC 2017 (AUC-0.918)
Xue et al.^127^, 2020	bkl/mel	ISIC 2017	Sen-0.750, Spe-0.934, AUC-0.932
Ding et al.^128^, 2021	bkl/mel	ISIC 2016, ISIC 2017	ISIC 2016 (Pre-0.719), ISIC 2017 (AUC-0.922)
Datta et al.^130^, 2021	HAM10000 (akiec/bcc/bkl /df/mel/nv/vasc), ISIC 2017 (bkl/nv)	HAM10000,ISIC 2017	HAM10000 (Acc-0.934, Pre-0.937, AUC-0.984), ISIC 2017 (Acc-0.904, Sen-0.916, Spe-0.833, AUC-0.959)
Alhudhaif et al.^131^, 2023	akiec/bcc/bkl/df/ mel/nv/vasc	HAM10000	Acc-0.959
Roy et al.^132^, 2024	akiec/bcc/bkl/df/ mel/nv/vasc	HAM10000	Acc-0.908, Pre-0.908, Sen-0.908, F1 score-0.912

**Observations:** Attention mechanisms are indeed an effective way to detect skin cancer and contribute to improved classification performance. However, they come with potential disadvantages. One significant challenge is the increased computational complexity introduced by attention mechanisms, leading to higher resource requirements during both training and inference. Additionally, attention-based models may be more susceptible to overfitting, particularly when dealing with limited datasets. The intricate nature of attention mechanisms can result in capturing noise and anomalies as if they were specific patterns, potentially impacting the model’s performance on unseen data^133^.

While the approaches demonstrated in^125,126^ are innovative, attention mechanisms that assign weights to relevant lesion areas have been shown to yield better results. Although Xue et al.^127^ proposes a novel approach, the sequential application of channel and spatial attention mechanisms may cause one to overshadow the other. Combining the attention maps first and then applying them to the features would ensure a balanced and simultaneous influence on feature refinement. While Ding et al.^128^ produce effective results using their novel DABN model, they do not provide results for multi-class classification. Notably, Refs.^130–132^ demonstrate exceptional effectiveness but face challenges in classifying cancer types with fewer samples. To address this issue, one possible mitigation approach can involve employing a combination of transfer learning with few-shot learning or zero-shot learning. Another potential solution can involve generating synthetic samples of the underrepresented classes using GAN-based models, providing a more sophisticated alternative to simple data augmentation techniques. Also, the combination of wavelet transform, soft attention, and SaFA module, in^132^, can be computationally intensive. Moreover, although attention mechanisms offer a degree of interpretability by emphasizing crucial parts of the input images, the specific interpretation of attention weights can sometimes be confusing. Comprehending the exact reasoning behind the model’s attention-based decisions may pose challenges.

##### Ensemble-based techniques

Ensemble techniques within DL consolidate predictions from multiple individual base models to generate more reliable predictions. By aggregating the knowledge from diverse models, ensemble methods commonly showcase enhanced adaptability to new data by diminishing errors in bias and variance. In the context of skin cancer, where lesion appearance can vary widely, ensemble techniques enable the system to recognize a broader range of features associated with different types of skin lesions. A comprehensive study of different methods for skin cancer classification using ensemble techniques is provided in Table 9. To facilitate a better generalization for the readers, we have adopted a broader interpretation of ensemble techniques, encompassing any combination of models, including strategies such as feature concatenation, fusion, and stacking, in addition to traditional ensemble approaches.

Harangi et al.’s study^134^ involved using various deep CNN architectures like AlexNet, VGGNet, and GoogLeNet. The final prediction was determined through a weighted majority vote, with each CNN’s vote being weighted by its confidence as indicated by the softmax output. Shahin et al.^135^ utilized two pre-trained deep CNN architectures, ResNet50 and InceptionV3, as distinct models in their ensemble. Instead of simply averaging the predictions from these individual models, the features extracted from both CNNs were concatenated and passed through a fully connected layer. Subsequently, the output of the fully connected layer was directed to a final layer equipped with a softmax activation function. Serte et al.^136^ introduced a Gabor wavelet^137^ based deep CNN. The approach involved decomposing input images into seven directional sub-bands. These seven sub-band images, in conjunction with the input image, acted as inputs for eight parallel CNNs, producing eight probabilistic predictions. The classification of skin lesions was accomplished through decision fusion using the sum rule. The Gabor-based strategy facilitated directional decomposition, allowing each sub-band to contribute isolated decisions that could be fused to enhance overall performance.

Aldwgeri et al.^138^ proposed an ensemble approach using multiple pre-trained CNN architectures like VGG16, ResNet50, InceptionV3, Xception, and DenseNet121. The predicted probabilities of each CNN for 7 different types of skin lesions were weighted and averaged to generate the final ensemble prediction. El-Katib et al.^139^ leveraged three pre-trained CNNs, GoogLeNet, ResNet101, and NasNetLarge for skin cancer classification. They combined the results from all the individual models into a global decision system based on a weighted approach, where each model’s weight was determined according to their individual accuracies. Bajwa et al.^140^ employed four deep CNN architectures, ResNet152, DenseNet161, SEResNeXt101 and NASNet to capture features from the skin lesion images focusing on aspects like color, texture, and borders. Predictions from the individual deep CNNs were not simply averaged. Instead, an ensemble learning approach was used to boost the accuracy and robustness of the model.

Gessert et al.^141^ utilized an ensemble of EfficientNet models for the classification of skin cancer on the imbalanced ISIC 2019 dataset. To address the challenge of class imbalance, they employed a loss balancing approach. This involved implementing a weighted cross-entropy loss function, where the weights assigned to classes were determined by their frequency in the training set. Imran et al.^142^ used an ensemble of three separate models, VGGNet, ResNet, and CapsNet. Here, predictions from each model were combined using majority voting, where the most frequent prediction becomes the final output. Hasan et al.^143^ introduced a hybrid CNN model comprising three distinct feature extractor modules, which are integrated to enhance the depth of feature maps for skin lesions. The fused feature maps undergo classification using separate fully connected layers, and their predictions are then ensembled to determine the lesion class. In the model’s architecture, FMG-1, FMG-2, and FMG-3 represent the three feature map generator modules. In the first level of ensembling, feature fusion is conducted through both channel averaging and channel concatenation. Ultimately, the output probability is determined by averaging the outputs of the fully connected layers, referred to as second level ensembling.

Ichim et al.^144^ examined two ensemble models. The first model consisted of three neural networks, MobileNet, DenseNet121, and DenseNet169, with an ensemble of individual decisions determined by the weights associated with each individual network. The second model incorporated two networks, MobileNet and DenseNet169, and followed a horizontal voting approach, where the ensembling was determined by the voting from the best models associated with the considered number of epochs. The second ensemble strategy was observed to deliver superior results compared to the first. Sarkar and Ray^145^ employed three deep CNN architectures, ResNet50, InceptionResNetV2, and DenseNet201, each of which was aided with an attention module. Subsequently, their prediction scores were combined using a novel classifier combination method based on Dempster–Shafer theory^146^. Ayesha et al.^147^ employed three pre-trained CNN models, VGG16, VGG19, and ResNet50, as feature extractors. The extracted features were concatenated into a composite feature vector, which was subsequently passed through a final dense layer for classification. Mandal et al.^148^ introduced a unique feature fusion method, combining the outcomes of two deep learning models. Their approach utilized Xception and Google’s Big Transfer (BiT-M) model as base learners, complemented by a squeeze and excitation attention module^149^ to improve the fused feature maps. This feature fusion network achieved an impressive accuracy of 79.50% on the challenging ISIC 2017 dataset.

Gairola et al.^150^ developed a deep network that leverages feature fusion to enhance skin cancer classification performance. The network features two main components: an improved single block (ISB) and an improved fusion block (IFB). The ISB increases the efficiency of a single CNN by enlarging the skin lesion feature map using zero padding, a convolutional layer, and ReLU activation. The IFB enhances the network’s capability by capturing extensive contextual information and global features through multi-dimensional exploration. They applied the ISB to enhance ResNet50 and ResNet101V2 architectures, combined their outputs, and utilized the IFB for the fusion and classification task. Naeem et al.^151^ employed borderline synthetic minority oversampling technique (SMOTE) to address class imbalance in skin cancer datasets. For feature extraction, they utilized both the Xception and ResNet101 models. The extracted features were then concatenated and passed through an additional convolutional layer. Afterwards, the feature map was flattened and used for classification to predict the skin cancer types.

**Table 9:** A comparative analysis of skin cancer classification methods using ensemble techniques.

Authors	Type	Dataset	Results
Harangi et al.^134^, 2018	bkl/mel/nv	ISIC 2017	Acc-0.838, AUC-0.848
Shahin et al.^135^, 2018	akiec/bcc/bkl/df/mel/nv/vasc	ISIC 2018	Acc-0.899, Pre-0.862, Sen-0.796
Serte et al.^136^, 2019	bkl/mel/nv	ISIC 2017	AUC-0.910
Aldwgeri et al.^138^, 2019	akiec/bcc/bkl/df/mel/nv/vasc	ISIC 2018	Acc-0.970, Sen-0.800, Spe-0.981
El-Khatib et al.^139^, 2020	mel/common nv	PH2	Acc-0.933, Sen-0.923, Spe-0.941
Gessert et al.^141^, 2020	akiec/bcc/bkl/df/mel/nv/vasc/scc	ISIC 2019	Acc-0.926
Bajwa et al.^140^, 2020	23 classes	DermNet	Pre-0.798, Sen-0.799, Spe-0.984
Imran et al.^142^, 2022	Malignant/benign	ISIC dataset	Acc-0.935, Pre-0.94, Sen-0.87, Spe-0.84, F1 score-0.92
Hasan et al.^143^, 2022	ISIC 2016 (mel/ nv), ISIC 2017 (bkl/mel/nv), ISIC 2018(akiec /bcc/bkl/df/ mel/nv/vasc)	ISIC 2016, ISIC 2017, ISIC 2018	ISIC 2016 (AUC-0.960), ISIC 2017 (AUC-0.950), ISIC 2018 (AUC-0.970)
Ichim et al.^144^, 2023	mel/non-mel	HAM10000	Acc-0.941, Pre-0.940, Sen-0.940, F1 score-0.940
Sarkar et al.^145^, 2023	akiec/bcc/bkl/df/mel/nv/vasc	HAM10000	Acc-0.932, Pre-0.931, Sen-0.932, F1 score-0.931
Ayesha et al.^147^, 2023	akiec/bcc/bkl/df/mel/nv/vasc	ISIC dataset	Acc-0.976, Pre-0.970, Sen-0.960, F1 score-0.960
Mandal et al.^148^, 2024	bkl/mel/nv	ISIC 2017	Acc-0.795, Pre-0.791, Sen-0.795, F1 score-0.792
Gairola et al.^150^, 2024	akiec/bcc/bkl/df/mel/nv/vasc	HAM10000	Acc-0.920, Pre-0.690, Sen-0.920, F1 score-0.730
Naeem et al.^151^, 2024	akiec/bcc/bkl/df/mel/nv/vasc/scc	10,284 images	Acc-0.982, Pre-0.983, Sen-0.984, F1 score-0.984, AUC-0.993

**Observations:** Ensemble methods involve training multiple models, and various strategies can be employed to combine their prediction scores, creating robust models without the need to repeatedly train individual base learners. However, this approach may introduce increased computational constraints and resource requirements as the number of base learners grows, potentially impacting deployment on real-time applications. Moreover, building and managing an ensemble also demands careful consideration of model selection, training, and integration, adding complexity and time consumption to the process.

Although Refs.^134,136,138–140,142,144^ produce impressive results, they employ simple majority voting, sum rule or weighted average based algorithms to combine the predictions from base learners. The implementation of more sophisticated combination algorithms for handling uncertain classes could potentially improve predictions. Additionally, in^136^, the Gabor wavelets employ parameters such as frequency and orientation that require meticulous tuning to achieve optimal performance. Gessert et al.^141^ introduces an innovative load balancing approach to address the class imbalance issue. Nonetheless, an enhanced ensembling strategy can further improve overall performance. References^135,143,148,150^ leverage the feature fusion strategy to produce enhanced feature maps with discriminative information, resulting in impressive test results. Optimizing feature selection before passing feature maps to the classification layer may further enhance performance. Sarkar et al.^145^ boasts remarkable results on the challenging HAM1000 dataset. However, the Dempster–Shafer theory-based combination introduces computational intensity, with complexity scaling up as the number of base learners increases. Ayesha et al.^147^ achieved promising results on the ISIC dataset; but, the authors did not evaluate their model on additional datasets, leaving its generalizability untested. Naeem and Anees^151^ demonstrates impressive results; however, the use of a proprietary dataset limits the ability to directly compare their findings with other studies.

#### Generative adversarial network-based techniques

The main utility of a GAN-based model lies in its capacity to generate synthetic samples that closely resemble real ones, preserving the same underlying data distribution^152^. Although GANs are not conventionally used directly for classification tasks such as skin cancer classification, they can indirectly contribute by addressing the imbalanced skin cancer datasets. GANs have the potential to generate synthetic images for underrepresented classes, thereby alleviating class imbalances and augmenting the dataset^153^. Table 10 presents a comprehensive overview of GAN-based techniques applied to skin cancer classification, detailing the diagnosed skin cancer types, datasets used and the achieved results.

**Fig. 9:**
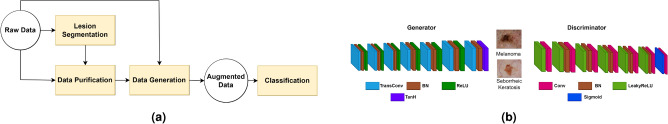
(**a**) Block diagram of the GAN-based model proposed by Bisla et al.^155^; (**b**) Architecture of the DCGAN proposed by Bisla et al.^155^.

Rashid et al.^154^ introduced a skin lesion classification system based on GANs. In their approach, they performed data augmentation on a training set of images by incorporating synthetic skin lesion images generated using a GAN. The generator module in their system employed a deconvolutional network, while the discriminator module used a CNN as the classifier. The CNN was trained to classify skin lesions into seven different categories. The proposed GAN-based approach outperformed both ResNet50 and DenseNet, achieving an accuracy of 86.10% for skin lesion classification. Bisla et al.^155^ introduced an approach that combines DL for data refinement and GANs for data augmentation. In their proposed framework, the initial step involved data purification using conventional image processing methods, followed by lesion segmentation utilizing a U-Net architecture. Subsequently, they employed decoupled deep convolutional GANs (DCGANs) to generate additional data. This refined and augmented dataset was then used to fine-tune a pre-trained ResNet50 model for the classification task, categorizing dermoscopic images into 3 types. Figure 9a,b depict the block diagram of this system and the architecture of the DCGAN model, respectively.

Chen et al.^156^ proposed a novel data augmentation approach for skin lesions employing a Self-Attention Progressive Generative Adversarial Network (PGAN) in their study. They employed stabilization techniques to enhance this generative model, resulting in an accuracy of 70.10%. Cheng et al.^208^ introduced a GAN architecture featuring multiple convolutional layers and upsampling in the generator module. Their discriminator module consisted of a CNN and a gradient penalty function, aimed at improving image quality and preventing artifacts.

**Table 10:** A comparative analysis of GAN-based skin cancer classification methods.

Authors	Type	Dataset	Results
Rashid et al.^154^, 2019	akiec/bcc/bkl/df/mel/nv/vasc	ISIC 2018	Acc-0.861
Bisla et al.^155^, 2019	bkl/mel/nv	ISIC 2017	Acc-0.861, AUC-0.915
Chen et al.^156^, 2022	akiec/bcc/bkl/df/mel/nv/vasc	ISIC 2018	Acc-0.700
Cheng et al.^208^, 2022	Malignant/benign	ISIC 2020	AUC-0.899

**Observations:** GAN-based models focus on creating synthetic data that mirrors the characteristics of a specified dataset. This principle holds significant promise in medical imaging, particularly in addressing the prevalent issue of limited data availability. However, in the context of skin cancer classification, the usefulness of GANs can be limited, as they often fail to address several domain-specific challenges. Skin lesions vary widely in size, shape, color, and texture, making it challenging for GANs to capture the subtle differences between benign and malignant lesions. These models often struggle with generating fine-grained details, such as irregular borders, asymmetry, and pigmentation variations, which are crucial for accurate diagnosis. GAN-generated images may appear blurry or overly smooth and lack the diagnostic precision needed. Additionally, the synthetic images might exhibit limited diversity and struggle to generalize effectively to new data, potentially causing overfitting. The training process for GANs can be computationally demanding and time-consuming, posing further challenges for their practical implementation in real-time clinical applications.

The outcomes of Refs.^154,156,208^ appear unsatisfactory, indicating the necessity for greater emphasis on enhancing the generator module’s effectiveness. Bisla et al.^155^ yields impressive results despite being a heavy network. They employed two separate DCGAN models to generate synthetic images for the underrepresented classes of melanoma and seborrheic keratosis in the ISIC 2017 dataset. Exploring the application of conditional deep convolutional GANs can help reduce computational constraints^157^.

#### Vision transformer-based techniques

ViTs have shown immense promise in various medical image analysis tasks, including skin cancer classification^158^. Their role in skin cancer classification involves leveraging their ability to learn representations from images broken down into patches and capture intricate patterns and features that distinguish among different types of skin lesions. Moreover, ViTs provide attention maps, highlighting areas, where the model focuses its attention^159^. In skin cancer classification, this can aid dermatologists in understanding which regions or features the model uses to make its predictions, contributing to interpretability. Table 11 provides a comprehensive list of ViT-based skin cancer classification techniques, highlighting the diagnosed skin cancer type, dataset, and the obtained results.

Aladhadh et al.^160^ designed a two-tier framework to classify skin cancer. In the first stage, they applied various data augmentation techniques to tackle class imbalance in the HAM10000 dataset. In the second stage, they employed a medical ViT, where the lesion images of size $72 \times 72$ were fed as input and each image was split into nine patches. Their transformer comprised three layers: an embedding layer, an encoder layer and a classifier layer. In the embedding layer, the transformer processed each patch as an individual token and then mapped it to a specific dimension with a learnable linear projection. The encoder layer contained self-attention and concatenation layers. The classifier layer predicted the final classification decision. Arshed et al.^161^, in their study, compared a fine-tuned ViT with various pre-trained CNN models of the ResNet, DenseNet and VGG families. According to their experiments, the ViT model outperformed all the other TL-based CNN models with an accuracy of 92.14%.

A four-block approach was proposed by Yang et al.^162^.It refers to the four-block architecture devised by their team. In the first block, seven different classes of cancer were balanced using various data augmentation methods. The image restructuring block formed the second block, which was responsible for splitting a 2D input image into a sequence of patches of the same size. These patches underwent flattening into tokens with consistent dimensions, followed by positional embedding to retain spatial information. The resulting output served as the input for the subsequent transformer encoder block. The third block comprised the transformer encoder block, featuring N repeated layers. Each layer contained a multi-head self-attention layer and a fully connected feed-forward network. The features derived from this encoder were employed for cancer classification in the final classification block. This block included a flatten layer, two normalization layers, a dense layer, and a softmax layer. This approach demonstrated promising results, surpassing other attention-based methods with an impressive classification accuracy of 94.10%. Krishna et al.^163^ leveraged ViT-based GANs (ViTGANs) to generate synthetic images as a solution to address the issue of class imbalance. Subsequently, they utilized a ViT consisting of identical layers of multi-head self-attention blocks and multi-layer perceptron (MLP) blocks to extract image features. These extracted features were then forwarded to a classifier for the estimation of class labels.

**Table 11:** A comparative analysis of ViT-based skin cancer classification methods.

Authors	Type	Dataset	Results
Aladhadh et al.^160^, 2022	akiec/bcc/bkl/df/mel/nv/vasc	HAM10000	Acc-0.961, Pre-0.960, Sen-0.965, F1 score-0.970
Arshed et al.^161^, 2023	akiec/bcc/bkl/df/mel/nv/vasc	HAM10000	Acc-0.921, Pre-0.926, Sen-0.921, F1 score-0.922
Yang et al.^162^, 2023	akiec/bcc/bkl/df/mel/nv/vasc	HAM10000, Dermofit	HAM10000 (Acc-0.941), Dermofit (Acc-0.805)
Krishna et al.^163^, 2023	akiec/bcc/bkl/df/mel/nv/vasc	HAM10000	Acc-0.974

**Observations:** ViTs, due to their built-in self-attention mechanisms, are experts in capturing global relationships among different parts of an image without the constraint of localized receptive fields. However, they are computationally intensive and consume high memory, especially as the image resolution increases. Since ViTs rely on understanding relationships between image patches, they benefit from large and varied datasets and may not generalize well if the images are limited or lack diversity. Moreover, ViTs process images in a sequence of non-overlapping patches, potentially losing detailed spatial information, crucial for precise lesion analysis^164^.

References^160–163^ demonstrate impressive results underscoring the efficiency of ViTs. Moreover, Yang et al.^162^ retains the spatial information by injecting additional positional embeddings into the tokens, allowing the model to learn and distinguish the position of tokens in the sequence. However, Krishna et al.^163^ demands substantial computational resources since they employ ViTGANs for image generation as well as ViTs for classification. Also, neither of these papers test their method on smaller datasets to validate the results.

#### Segmentation-guided classification techniques

In the context of skin cancer classification, segmentation-guided classification techniques serve to be extremely powerful. It helps isolate the lesion from the surrounding skin and other artifacts in the image. This reduction in noise and background interference leads to a cleaner input for the classification model, potentially improving its performance. These techniques enhance the extraction of features specifically from the identified ROI. This results in a more precise representation of the skin lesion, allowing the classification model to focus solely on relevant information. Table 12 provides a comprehensive list of various segmentation-guided skin cancer classification techniques.

Yu et al.^165^ introduced a two-stage framework for melanoma detection. The first stage involved lesion segmentation, where a fully convolutional residual network (FCRN) with 16 residual blocks was employed to accurately delineate the skin lesion from the surrounding healthy skin. This ensured that the subsequent classification focused specifically on the relevant region. In the second stage, a distinct ResNet architecture was utilized to classify the segmented lesion as either melanoma or non-melanoma. The melanoma classification achieved an accuracy of 85.50% with segmentation and 82.80% without segmentation on the ISIC 2016 dataset. The framework proposed by Al-masni et al.^166^ integrated two key stages: a skin lesion boundary segmentation stage and a multiple skin lesion classification stage. Initially, skin lesion boundaries were segmented from dermoscopy images using a full resolution convolutional network (FrCN). Subsequently, various deep CNNs, including Inceptionv3, ResNet50, InceptionResNetv2, and DenseNet201, were employed for the classification of the segmented skin lesions. The first stage, accomplished by FrCN, was crucial as it extracted prominent features essential for diagnosing various types of skin lesions. The selection of a promising classifier was determined through thorough testing of various CNNs.

Hasan et al.^167^ proposed the Dermo-DOCTOR system, utilizing end-to-end dual encoders for both segmentation and classification tasks. The model incorporated two distinct encoders, each specialized in extracting different features from input images. Encoder 1 focused on global features, capturing the overall structure and shape of the lesion, while encoder 2 concentrated on local features, extracting fine-grained details within the lesion. These encoders were seamlessly integrated into a single, end-to-end trainable architecture, enabling simultaneous detection and recognition. The features extracted from both encoders were fused and directed into two separate branches: the detection branch, responsible for localizing the precise boundaries of the lesion within the image, and the recognition branch, which classified the lesion into different categories. Gerges et al.^168^ employed a segmentation strategy, utilizing the k-means clustering algorithm, with a k-value of 2, for ROI extraction. The resultant segmented images were then passed as input to a CNN consisting of 2 convolutional layers, each succeeded by a pooling layer, and concluded with 2 fully connected output layers.

Sai Charan et al.^169^ employed a two-path CNN model, incorporating two separate deep CNNs. One CNN received original images as input, while the other received images segmented using the U-Net architecture. Deep features from both CNNs were combined and utilized by the dense layers for the classification process. Gururaj et al.^170^ employed an encoder-decoder architecture for image segmentation, incorporating convolutions and max pooling in the encoder, and upsampling along with convolutions in the decoder. The encoder’s role was to identify and capture pertinent patterns, textures, and structures within skin lesions, with deeper layers gradually learning more intricate representations. Meanwhile, the decoder played a critical role in precisely localizing and delineating lesion boundaries, refining the features extracted by the encoder and generating a detailed and pixel-wise segmentation map. For the classification task, the study utilized two deep CNNs, namely DenseNet169 and ResNet50. In this task, DenseNet169 outperformed ResNet50, achieving an impressive accuracy of 91.20% on the HAM10000 dataset.

Khan et al.^171^ proposed a framework consisting of two main blocks: one for segmentation and localization and another for classification. For the lesion segmentation task, they employed two separate CNNs. The original images were fed into one CNN, while contrast-enhanced images were fed into the other. The outputs from these CNNs were then fused using the joint probability distribution and marginal distribution function to create a refined segmented image. This refined image was subsequently used as input for a 30-layer CNN architecture, which included 2 fully connected layers. Features extracted from these layers were combined using summation discriminant correlation analysis. To prevent feature redundancy, the regula falsi method was utilized for dimensionality reduction. Finally, the selected features were classified using an ELM classifier.

Similar to the approach of Gerges et al.^168^, Naeem et al.^172^ applied the k-means clustering algorithm with two clusters to segment the ROI from lesion images. Their methodology first employed anisotropic diffusion to denoise the images, followed by the application of SMOTE-Tomek to address the class imbalance problem in the ISIC 2019 dataset. After pre-processing, segmentation was performed, and feature extraction was conducted using both VGG19 and HOG. The extracted features were then serially fused, and maximum entropy-based feature selection was applied to retain the most informative features. Finally, the selected feature vector was fed into a classification head to generate predictions for skin cancer classification.

**Table 12:** A comparative analysis of segmentation-guided skin cancer classification methods.

Authors	Type	Dataset	Results
Yu et al.^165^, 2016	mel/non-mel	ISIC 2016	Acc-0.855
Al-masni et al.^166^, 2020	ISIC 2016 (mel/nv), ISIC 2017 (bkl/mel/nv), ISIC 2018 (akiec/bcc/bkl/df/mel/nv/vasc)	ISIC 2016, ISIC 2017, ISIC 2018	ISIC 2016 (Acc-0.818, Sen-0.818, F1 score-0.826), ISIC 2017 (Acc-0.816, Sen-0.753, F1 score-0.756), ISIC 2018 (Acc-0.893, Sen-0.810, F1 score-0.813)
Hasan et al.^167^, 2021	bkl/mel/nv	ISIC 2017	Acc-0.780, Pre-0.790, Sen-0.780
Gerges et al.^168^, 2021	Malignant/benign	MED-NODE	Acc-0.970
Sai Charan et al.^169^, 2022	akiec/bcc/bkl/df/mel/nv/ vasc/scc	ISIC 2019	Acc-0.886
Gururaj et al.^170^, 2023	akiec/bcc/bkl/df/mel/nv/ vasc	HAM10000	Acc-0.912, F1 score-0.917
Khan et al.^171^, 2024	akiec/bcc/bkl/df/mel/nv/ vasc	HAM10000	Acc-0.870, Sen-0.869
Naeem et al.^172^, 2024	akiec/bcc/bkl/df/mel/nv/ vasc/scc	ISIC 2019	Acc-0.983, Pre-0.982, Sen-0.982, F1 score-0.982

**Observations:** Segmentation provides information about the spatial extent and boundaries of the skin lesions. This can be valuable for especially understanding the localized characteristics of the skin condition, aiding in more accurate classification. While segmentation-guided classification techniques are immensely promising, they also come with certain limitations. The need for precise segmentation may lead to increased resource requirements, making the techniques computationally intensive. Additionally, errors in the segmentation process can propagate into the subsequent classification stage, affecting the overall accuracy of the system.

While Refs.^165–167,169–171^ demonstrate impressive results, the utilization of separate networks for segmentation and classification introduces increased model complexity, making training and optimization more challenging. Additionally, comprehending the contributions of features from each encoder in^167^ and their impact on final decisions may pose challenges. The FrCN block presented in^166^, is adept at pixel-wise classification and can generate precise segmentation masks. However, its computational cost is high, primarily attributed to pixel-level computations. The framework described in^171^ relies on image quality, including contrast enhancement. As a result, it may be sensitive to variations in image acquisition conditions, such as differences in lighting and resolution. References^168,172^ utilize a rather simple and computationally efficient segmentation strategy based on the k-means clustering algorithm. However, they do not provide an explicit justification for choosing a k-value of 2. Furthermore, the reliance of Gerges and Shih^168^ on the small MED-NODE dataset for testing prompts inquiries about the model’s generalizability to larger datasets. Although Naeem and Anees^172^ demonstrates impressive performance on the ISIC 2019 dataset, their model’s dependence on manually designed feature extraction methods may limit its adaptability in real-world settings, especially when compared to fully end-to-end DL approaches.

### Hybrid techniques

The combination of DL and ML techniques (hybrid approaches) holds significant importance for image classification tasks. DL methods, especially pre-trained CNN models, excel in feature extraction from images. When coupled with traditional ML algorithms like SVM or RF, these hybrid models can leverage the strengths of both approaches, potentially leading to improved classification accuracy. Moreover, DL models often demand abundant labelled data for training. Hybrid strategies can alleviate this requirement by leveraging pre-trained DL models for feature extraction, followed by employing ML techniques on these extracted features^173^. In the realm of skin cancer classification, these hybrid techniques prove highly beneficial. DL models are adept at learning hierarchical representations from raw data, which can be beneficial for capturing intricate patterns from skin lesion images. By integrating ML algorithms, the hybrid models can utilize these patterns as input features, facilitating accurate classification, even in scenarios of limited data, a common challenge in skin cancer applications. Additionally, combining features extracted by DL models with handcrafted features can enhance the robustness of the classification process. This approach leverages the complementary strengths of both feature types; while DL models capture complex patterns, handcrafted features can provide contextual or domain-specific insights that may improve classification performance. By incorporating both types of features, hybrid models can create a more comprehensive representation of the data, ultimately leading to improved diagnostic accuracy and reliability in clinical settings. A comprehensive list of various skin cancer classification systems based on hybrid techniques is listed in Table 13.

Shoieb et al.^174^ developed a standard CNN for feature extraction. The CNN consisted of convolution, pooling, non-linear and fully connected layers. Initially, the first convolutional layer was dedicated to capturing rudimentary features such as edges and corners, while subsequent convolutional layers focused on extracting more intricate patterns. The pooling layers condensed the representations of these features. The extracted features were then trained on a linear SVM, which executed classification by determining the hyperplane that maximized the margin between the two classes (melanoma and non-melanoma). Dorj et al.^175^ employed a pre-trained AlexNet model for feature extraction from dermoscopic images and fine-tuned the final layers of the model. Then, they leveraged an error-correcting output codes (ECOC) SVM classifier^176^ for the classification task. ECOC method converts a problem of classifying among multiple classes into a series of two-class classification problems (one-vs-all approach).

Khan et al.^177^ utilized pre-trained ResNet50 and ResNet101 to extract diverse features from the dermoscopic lesion images, focusing on textures and borders. These extracted features from both the deep CNNs were fused to create a comprehensive lesion representation. Then, they employed the kurtosis controlled PCA (KcPCA)^178^ method to select discriminative features based on high kurtosis from the fused representation. Finally, an SVM with an RBF kernel was used to classify the selected features into distinct skin lesion categories. This work aimed to enhance classification accuracy by leveraging distinct feature extraction, fusion, selection, and classification techniques. Mahbod et al.^179^ introduced a methodology for extracting deep features using pre-trained CNNs, including AlexNet, ResNet18, and VGG16, for skin lesion classification. These pre-trained networks served as deep-feature generators, and the extracted features were used to train a multi-class SVM classifier. The classification results from the SVM were then combined for the final classification.

Mahbod et al.^180^ also employed 3 sets of CNNs with different architectures. Set 1 consisted of two identical ResNet50 networks, set 2 consisted of two identical EfficientNetB0 networks, and set 3 consisted of two identical EfficientNetB1 networks. Each set of CNNs shared the same architecture, but different fine-tuning strategies were applied to each set. The CNNs within each set extracted features from the images, resulting in multiple sets of feature vectors. These feature vectors were concatenated to create a comprehensive feature representation for each image. The fused feature vectors were then input into different SVMs, each trained for a specific lesion category. This approach allowed for separate classifiers targeting different lesions, potentially enhancing performance. Each SVM produced a class probability vector, and these vectors were averaged to generate the final ensemble prediction probabilities. Kassem et al.^181^ utilized GoogLeNet for feature extraction from the lesion images. They opted to remove only the last two layers, retaining the original fully connected layers within the GoogLeNet architecture for feature extraction. The extracted features were then employed in a multi-class SVM for classification.

Benyahia et al.^182^ employed 17 pre-trained CNN architectures as feature extractors to capture different aspects of the lesion like textures, borders, and color patterns. Subsequently, they utilized various ML classifiers to classify the lesion images. In their work, a combination of DenseNet201 and k-NN yielded the best results on the challenging ISIC 2019 dataset. 8 pre-trained CNN architectures, VGG16, VGG19, ResNet50, ResNet101, InceptionV3, DenseNet121, MobileNet, and Xception were used to extract deep features from dermoscopic lesion images by Gajera et al.^183^. They used SVM as a classifier to train the extracted features from all the deep CNNs, out of which DenseNet121 yielded the highest accuracy for melanoma detection.

**Fig. 10:**
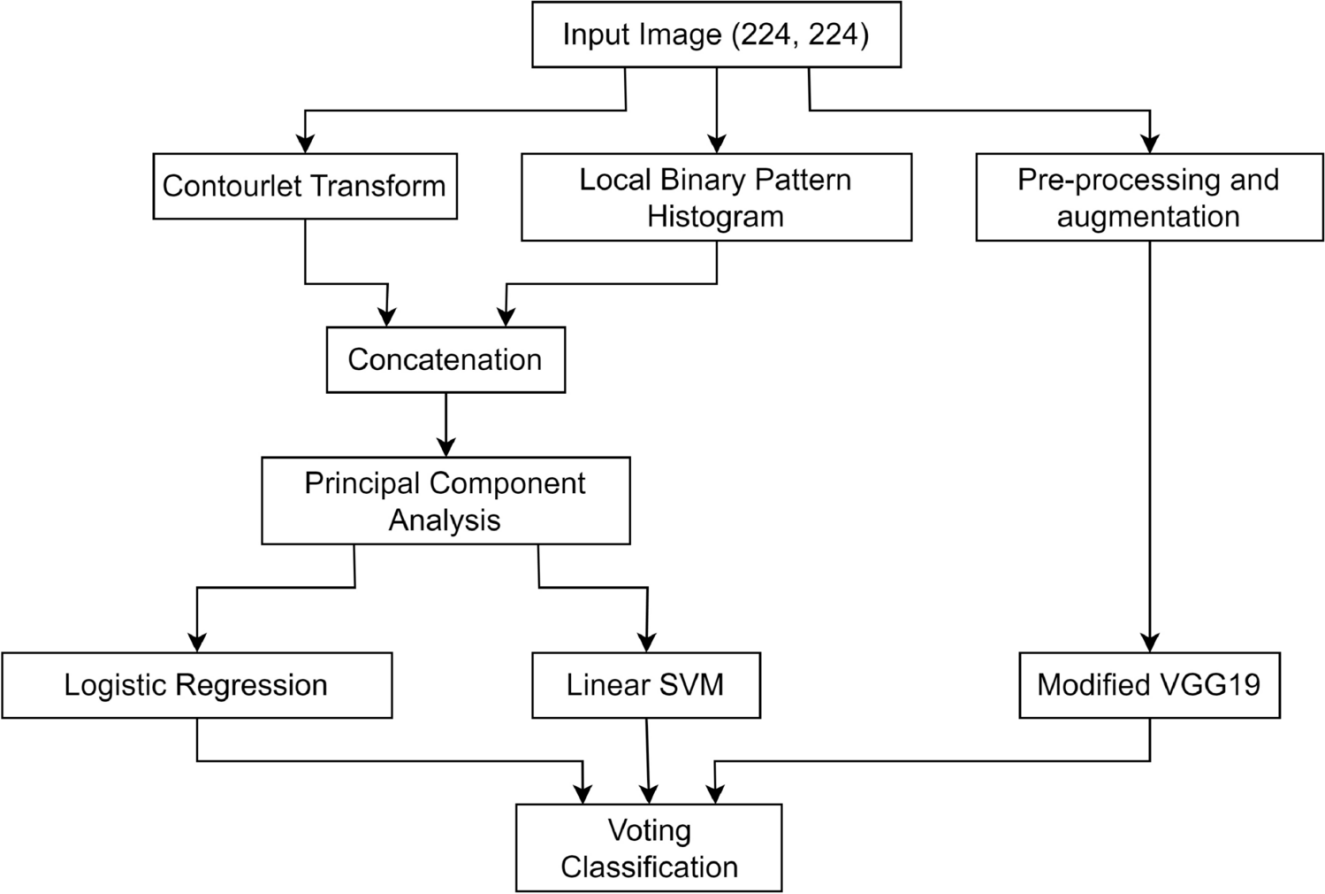
Overview of the hybrid model proposed by Tembhurne et al.^184^.

Tembhurne et al.^184^ proposed a multi-branch approach by combining ML and DL techniques for skin lesion classification. In the DL branch, they employed a pre-trained VGG16 network to extract high-level features and perform image classification. In the ML branch, they leveraged the contourlet transform technique^185^ and LBPH to extract texture and color features from the image. These features were then concatenated, subjected to dimensionality reduction via PCA, and fed into two ML models-logistic regression and linear SVM. The final classification was determined by combining the outcomes from both branches through a voting mechanism, resulting in the categorization of images as malignant or benign. Figure 10 depicts the overview of this model. Keerthana et al.^186^ utilized a hybrid CNN architecture of DenseNet201 and MobileNet to capture both low-level features like textures and edges and high-level features like lesion patterns and shapes. Then, they applied PCA to reduce the dimensionality of the extracted features and improve computational efficiency. Finally, they leveraged an SVM classifier trained on the set of features with reduced dimensionality. Similar to the approach by Tembhurne et al.^184^, Naeem et al.^187^ also employed two branches that integrated both ML and DL techniques for feature extraction. In the ML branch, they utilized histograms for extracting color features, the GLCM for capturing global textural information, the features from accelerated segment test (FAST) and rotated binary robust independent elementary features (BRIEF) descriptors^188^ for local textural information, and Zernike moments^189^ to extract shape features. In the DL branch, they used the InceptionV3 model as the feature extractor. The features from both branches were then fused using an entropy-based fusion method, similar to the technique described in^172^, and the fused features were passed through the final dense layers for classification.

**Table 13:** A comparative analysis of skin cancer classification methods using hybrid techniques.

Authors	Type	Dataset	Results
Shoieb et al.^174^, 2016	mel/non-mel	DermIS, DermQuest	DermIS (Acc-0.938), DermQuest (Acc-0.941)
Dorj et al.^175^, 2018	akiec/bcc/mel/scc	3753 images	Acc-0.960
Khan et al.^177^, 2019	Malignant/benign	HAM10000, ISIC 2016, ISIC 2017	HAM1000 (Acc-0.898), ISIC 2016 (Acc-0.902), ISIC 2017 (Acc-0.956)
Mahbod et al.^179^, 2019	bkl/mel	ISIC 2017	AUC (bkl: 0.976, mel: 0.838)
Mahbod et al.^180^, 2019	bkl/mel	ISIC 2017	AUC (bkl: 0.956, mel: 0.873)
Kassem et al.^181^, 2020	akiec/bcc/bkl/df/ mel/nv/vasc/scc	ISIC 2019	Acc-0.942, Pre-0.736, Sen-0.745, Spe-0.965, F1 score-0.740
Benyahia et al.^182^, 2022	ISIC 2019 (akiec/ bkl/bcc/df/nv/ mel/vasc/scc), PH2 (mel/non-mel)	ISIC 2019, PH2	ISIC 2019 (Acc-0.923), PH2 (Acc-0.990)
Gajera et al.^183^, 2023	mel/non-mel	HAM10000, ISIC 2016, ISIC 2017, PH2	HAM10000 (Acc-0.810), ISIC 2016 (Acc-0.805), ISIC 2017 (Acc-0.812), PH2 (Acc-0.983)
Tembhurne et al.^184^, 2023	Maligant/benign	ISIC archive	Acc-0.930, Sen (malignant: 0.860, benign: 0.997)
Keerthana et al.^186^, 2023	mel/non-mel	ISIC 2016	Acc-0.880
Naeem et al.^187^, 2024	akiec/bcc/bkl/df/ mel/nv/vasc/scc	ISIC 2019	Acc-0.978, Pre-0.983, Sen-0.979, F1 score-0.981, AUC-0.997

**Observations:** Hybrid approaches are beneficial since they eliminate the need for manual feature engineering, as they automatically learn relevant representations, spatial relationships and local structures from the data using DL models, reducing human bias and effort. Additionally, this approach proves advantageous, particularly in scenarios with limited labelled data, reducing computational demands without compromising performance. Furthermore, computational efficiency can be enhanced by reducing the dimensionality of features extracted by DL models before feeding them into ML classifiers. However, this reduction may result in the loss of crucial information. Therefore, maintaining a balance between dimensionality reduction and information preservation is vital. Consequently, optimal feature selection becomes imperative before inputting these extracted features into the ML classifier. This step aids in identifying the most relevant and discriminative features, improving classification performance and reducing the risk of overfitting.

The simple network by Shoieb et al.^174^ achieves fairly decent results on older datasets but its performance remains untested on newer, more complex datasets. Dorj et al.^175^ relies on a proprietary dataset for testing, limiting comparative studies with other models. References^179,180^ do not report overall accuracy scores making it difficult for comparison with other related methodologies. While the approach demonstrated in^180^ is robust, the use of separate SVMs for each class may not fully exploit potential correlations between classes. References^177,183,184^ showcase fairly decent results on challenging datasets. However, they do not perform multi-class classification, which could have been more relevant in real-world scenarios. Leveraging PCA to reduce the dimensionality of features, Keerthana et al.^186^ demonstrates computational efficiency without compromising classification accuracy. The extensive experiments performed by Benyahia et al.^182^ highlight the superiority of combining DenseNet201 and k-NN over using only DenseNet201. Similarly, in Kassem et al.^181^, the results indicate that a combination of GoogLeNet and SVM surpasses the performance of using only GoogLeNet. Naeem et al.^187^ highlights the significance of integrating both ML and DL techniques for feature extraction. However, the evaluation of their model is limited to a subset of the ISIC 2019 dataset, rather than the full dataset, which may restrict the comprehensiveness of their findings. These studies further validate the significance of using hybrid approaches in skin cancer classification.

### Multimodal techniques

Multimodal techniques in skin cancer classification combine varied data streams, including images, clinical data, and pathology reports, providing a comprehensive and robust diagnostic solution. These approaches enhance generalization and improve diagnostic confidence by capturing multifaceted patterns. Integrating complementary information enhances interpretability, fostering a nuanced comprehension of skin lesions. A comprehensive study of different methods for skin cancer classification using such approaches is provided in Table 14.

Yap et al.^190^ introduced a multimodal fusion model which combined information from three modalities: macroscopic image, dermoscopic image, and patient metadata. The macroscopic images are analyzed by a CNN for spatial features and the dermoscopic images are analyzed by a different CNN for finer details. Patient metadata is encoded with a separate network using an embedding layer. The extracted features are fused and fed into a final neural network classifier to categorize the lesion images into malignant and benign. Ou et al.^191^ developed a DL model employing 2 encoders to extract information from image data and metadata. The image encoder utilized a deep CNN for feature extraction from the images, while the meta encoder processed textual metadata, including patients’ attributes and lesion characteristics, using an MLP. Subsequently, a multimodal fusion module with intra-modality self-attention and inter-modality cross-attention was employed to highlight crucial regions within each modality and capture interactions between image and metadata features respectively. The final classification layer predicted 6 distinct types of lesions.

Tajjour et al.^192^ introduced a multimodal network using an ensemble of CNN and MLP. They used a CNN to analyze the original RGB image to extract high-level features related to lesion shape, texture, and borders and used an MLP to process patients’ metadata and features, extracted from different color spaces, to capture additional information on color distribution, illumination, and energy within the lesion. These features were fused and classified using a final classification layer. The results revealed a top-1 accuracy of 86% and a top-2 accuracy of 95% for the seven classes. Omeroglu et al.^193^ introduced a multi-branch structure for multi-label skin lesion classification. They used two branches in their feature extraction phase, a dermoscopy branch and a clinical branch. A modified Xception architecture was used to extract visual features in the dermoscopy branch, whereas, they processed clinical data into numerical representations in the clinical branch. They also employed a soft attention module to analyze feature maps from both branches and assign weights based on their relevance to specific lesions. Subsequently, they designed a hyperbranched fusion block to combine weighted feature maps from different scales within each branch and across branches, creating a richer and more comprehensive representation of the lesion. Finally, they used a multi-label classification layer to compute output probabilities for each possible skin lesion label.

SM et al.^194^ employed EfficientNetB6 as the backbone of their model to extract features related to melanoma and non-melanoma lesions. They also designed a simple neural network to train the contextual information given in the ISIC 2020 dataset. The extracted features from both networks were concatenated and trained using a light gradient boosting machine (LGBM) classifier. In addition to this, they also utilized the Ranger optimizer^195^ to improve the model’s generalizability and overall performance. Kumar et al.^196^ introduced a multimodal network that utilizes handcrafted features derived from different domains of lesion images: spatial, frequency, and cepstrum. Initially, the RGB images are converted to grayscale. For the frequency domain, spectrograms are calculated, while for the cepstrum domain, cepstral coefficients are computed. The grayscale images, spectrograms, and cepstral coefficients are then transformed from 2-D to 1-D features. These features are concatenated and passed as input into a 1-D multi-headed CNN comprising three heads. The outputs from these heads are then concatenated for the classification task.

Sahoo et al.^197^ introduced an innovative multimodal framework for skin cancer classification by integrating deep features with wavelet features. They utilized a pre-trained ResNet50 model to extract deep features from lesion images. These images were then transformed into the wavelet domain using the lifting wavelet transform (LWT)^198^, specifically utilizing the level-2 approximation component as the wavelet features. The deep and wavelet features were combined and then subjected to the neighborhood component analysis (NCA) algorithm^199^ to select a reduced subset of the fused features. This reduced feature set was finally classified using an MLP.

**Table 14:** A comparative analysis of skin cancer classification methods using multimodal techniques.

Authors	Type	Dataset	Results
Yap et al.^190^, 2018	mel/non-mel	ISIC 2017	AUC-0.858
Ou et al.^191^, 2022	akiec/bcc/bkl/mel/nv/scc	2298 cases	AUC-0.947
Tajjour et al.^192^, 2023	akiec/bcc/bkl/df/mel/nv/vasc	HAM10000	Top-1 acc-0.86, Top-2 acc-0.95, Pre-0.870, Sen-0.860, F1 score-0.860, AUC-0.960
Omeroglu et al.^193^, 2023	multi-label	ISIC 2016	Acc-0.830
SM et al.^194^, 2023	mel/non-mel	ISIC 2020	AUC-0.968
Kumar et al.^196^, 2024	HAM10000 (akiec/bcc/bkl/df/mel/nv/vasc), DermNet (akiec/ep/mel/nf/bkl/uh/vasc)	HAM10000, DermNet	HAM10000 (Acc-0.897, Pre-0.890, Sen-0.892, Spe-0.927, F1 score-0.891, AUC-0.934), DermNet (Acc-0.886, Pre-0.888, Sen-0.883, Spe-0.911, F1 score-0.880, AUC-0.930)
Sahoo et al.^197^, 2024	mel/non-mel	ISIC 2016, PH2	ISIC 2016 (Acc-0.781, Sen-0.780, Spe-0.750, AUC-0.806), PH2 (Acc-0.980, Sen-1.000, Spe-0.969, AUC-0.996)

**Observations:** While multimodal approaches enhance overall reliability and generalizability, such approaches introduce complexities due to potential challenges in aligning diverse data sources. Additionally, data collection for multiple modalities can be more resource-intensive and expensive.

Multimodal strategies demonstrated in^190–194,196,197^, offer new perspectives. However, Yap et al.^190^ falls short of achieving high performance, and Tajjour et al.^192^ does not report overall accuracy, limiting comparative analyses with other work. Ou et al.^191^ introduces an innovative multimodal fusion strategy but uses a self-procured dataset for testing instead of standard datasets. SM et al.^194^ demonstrates impressive results on the demanding ISIC 2020 dataset. Nevertheless, it falls short by not addressing multi-class classification, which could be more pertinent in real-world scenarios. Although Omeroglu et al.^193^ introduces a novel multi-branch structure, its multi-label classification approach limits direct comparisons with similar work. This method can be investigated further on more recent datasets for multi-class classification. Kumar et al.^196^ yields impressive results on challenging datasets but relies on manually engineered features from different domains of lesion images. They neither explicitly mention why they have specifically used the frequency and cepstrum domains, in addition to the spatial domain, nor how the features extracted from these domains could help boost classification performance. While Sahoo et al.^197^ demonstrates exceptional results on older datasets, it does not provide results on newer, more complex datasets.

## Performance metrics

Performance metrics play a critical role in gauging the effectiveness and utility of a model. These metrics are instrumental in determining a model’s ability to accurately predict the appropriate type or label for an input in classification tasks. In this section, we explore the commonly employed evaluation metrics utilized for skin cancer classification tasks. Before delving into the discussion on these metrics, it’s essential to familiarize ourselves with the subsequent terms:

- **True positive (TP):** This refers to the number of instances that are actually positive and are correctly predicted as positive by the model. In a medical context, it would be when the model correctly identifies a patient as having a particular condition or disease.
- **False positive (FP):** This refers to the number of instances that are actually negative but are incorrectly classified as positive by the model. In a medical scenario, it would mean the model falsely indicates a patient as having a condition when they don’t.
- **False negative (FN):** This refers to the number of instances that are actually positive but are incorrectly predicted as negative by the model. In a medical context, it would mean the model fails to identify a patient who actually has a condition, falsely indicating them as healthy.
- **True negative (TN):** This refers to the number of instances that are actually negative and are correctly predicted as negative by the model. In a medical setting, it would mean the model correctly identifies a healthy individual as not having a particular condition.

### Accuracy (Acc)

Accuracy^200^ computes the proportion of accurately predicted samples, encompassing both true positives and true negatives ($TP+TN$), relative to the total sample size. It assesses the overall correctness of the model’s predictions. Equation (2) denotes the formula for accuracy.

2 $$\begin{aligned} Acc = \frac{TP + TN}{TP + FP + FN + TN} \end{aligned}$$

### Precision (Pre)

Precision^200^ quantifies the proportion of accurately predicted positive observations (*TP*) relative to all predicted positive instances ($TP+FP$). It measures the accuracy of positive predictions, indicating the relevance of selected items within the predictions. Equation (3) denotes the formula for precision.

3 $$\begin{aligned} Pre = \frac{TP}{TP+FP} \end{aligned}$$

### Sensitivity (Sen)

Sensitivity^200^, also known as recall, measures the ratio of correctly predicted positive observations (*TP*) concerning all actual positive instances ($TP+FN$). This metric signifies the model’s capability to recognize all relevant instances, assessing its completeness in identification. Equation (4) denotes the formula for sensitivity.

4 $$\begin{aligned} Sen = \frac{TP}{TP + FN} \end{aligned}$$

### Specificity (Spe)

Specificity^200^ computes the proportion of accurately predicted negative observations (*TN*) in relation to all actual negative instances ($TN+FP$). This metric evaluates the model’s capability to accurately recognize negative instances. Equation (5) denotes the formula for specificity.

5 $$\begin{aligned} Spe = \frac{TN}{TN + FP} \end{aligned}$$

### F1 score

F1 score^200^ represents the harmonic mean of precision and recall, amalgamating both metrics into a unified measure. By striking a balance between precision and recall, it offers a singular score that comprehensively considers both false positives and false negatives. Equation (6) denotes the formula for F1 score.

6 $$\begin{aligned} F1\;score = 2\times \frac{(Precision\times Recall)}{(Precision+Recall)} \end{aligned}$$

### Receiver operating characteristic (ROC) curve

The ROC curve^201^ graphically depicts how well a classification model performs across a range of thresholds. By plotting the true positive rate (*sensitivity*) against the false positive rate ($1-specificity$) across various thresholds, each point on the curve reflects a *sensitivity*/*specificity* pair linked to a specific threshold setting. A superior model showcases a ROC curve closer to the top-left corner, signalling increased sensitivity and specificity across different threshold values.

### Area under the curve (AUC)

The AUC^201^ metric measures the total area enclosed by the ROC curve, summarizing a classification model’s performance across all feasible thresholds. A higher AUC value, closer to 1, signifies the model’s enhanced ability to differentiate between classes. An AUC of 0.5 indicates random chance, while an AUC of 1 represents a perfect classifier.

## Loss functions

Loss functions serve a pivotal role in optimizing the parameters and monitoring the training progress of supervised models. This segment explores commonly employed loss functions in the classification of skin cancer as found in the literature.

### Binary cross-entropy loss

Binary cross-entropy loss^202^, also known as log loss, is a commonly applied loss function in binary classification tasks, such as in skin cancer classification, aiming to distinguish between two classes (e.g., benign and malignant lesions). It penalizes the model based on the difference between predicted probabilities and actual binary labels. As the predicted probability diverges from the true label, the loss increases significantly. During training, the goal is to minimize the average binary cross-entropy loss across the entire dataset by adjusting the model’s parameters (weights and biases). Equation (7) denotes the formula for binary cross-entropy, where *N* represents the number of samples, $y_i$ denotes the true binary label for sample *i*, and $p_i$ is the predicted probability for sample *i*.

7 $$\begin{aligned} L_{BCE} = - \frac{1}{N} \sum _{i=1}^{N} \left[ y_i \cdot \log (p_i) + (1 - y_i) \cdot \log (1 - p_i) \right] \end{aligned}$$

### Categorical cross-entropy loss

Categorical cross-entropy loss serves as a prevalent loss function applied in scenarios involving the identification of multiple classes of skin lesions, as seen in multi-class classification tasks. This function determines loss by contrasting the predicted probabilities associated with each class against the actual labels for each sample within the dataset. The magnitude of penalization escalates to substantial disparities between the predicted and true probabilities. It penalizes the model more for larger differences between the predicted and true probabilities. During model training, the objective is to minimize the average categorical cross-entropy loss across all classes, optimizing the model’s parameters to improve classification accuracy. Equation (8) represents the formula for categorical cross-entropy, where *N* represents the number of samples, *C* is the number of classes, $y_{ij}$ and $p_{ij}$ denote the true label (0 or 1) and the predicted probability, respectively for sample *i* and class *j*.

8 $$\begin{aligned} L_{CCE} = - \frac{1}{N} \sum _{i=1}^{N} \sum _{j=1}^{C} y_{ij} \cdot \log (p_{ij}) \end{aligned}$$

### Weighted loss

Weighted loss functions^203^ are modifications applied to standard loss functions, such as cross-entropy loss. In skin cancer classification, certain rare types of lesions might have fewer samples compared to others. These weighted loss functions aim to rectify this imbalance by assigning weights to each class based on their frequency or significance. Higher weightage is given to less frequent or critical classes while reducing the emphasis on more common classes. By incorporating these weights into the standard loss function, the model accentuates its attention on classes that are underrepresented during the training phase. This adjustment in the contribution of each class to the overall loss enables the model to prioritize learning from the less represented classes, thereby refining its comprehension of these infrequent lesion types. In the context of cross-entropy loss for multi-class classification, the weighted loss can be represented as Equation (9), where $w_j$ stands for the weight assigned to class *j*. Another example of a weighted loss function is the margin-aware adaptive-weighted (MAAW) loss, introduced by Debasmit et al.^204^. In this method, the weight adjusts dynamically for each minibatch, relying on the inverse class frequencies.

9 $$\begin{aligned} L_{weighted} = - \frac{1}{N} \sum _{i=1}^{N} \sum _{j=1}^{C} w_j \cdot y_{ij} \cdot \log (p_{ij}) \end{aligned}$$

### Focal loss

Focal loss^205^ is a specialized loss function primarily designed to address the issue of class imbalance in image classification tasks. In skin cancer classification, datasets often exhibit an imbalance, where one class might be significantly smaller in quantity compared to others. Focal loss aims to mitigate the influence of predominant classes by reducing the impact of well-classified instances and placing greater emphasis on challenging samples. Focal loss introduces an adjustment factor that diminishes the contribution of easily classified examples to the loss function while emphasizing the misclassified or challenging samples. Equation (10) signifies the formula for focal loss, where *N* represents the number of samples, $p_{ti}$ denotes the predicted probability for the true class label of sample *i*, and $\gamma$ is the tunable focusing parameter, which can be adjusted to control the degree of emphasis on difficult samples versus easy ones during training. The term $(1 - p_{ti})^\gamma$ dynamically adapts the loss contribution, placing greater emphasis on challenging examples by diminishing the effect of well-classified instances.

10 $$\begin{aligned} L_{focal} = - \frac{1}{N} \sum _{i=1}^{N} \left[ (1 - p_{ti})^\gamma \cdot \log (p_{ti}) \right] \end{aligned}$$

### Triplet loss

Triplet loss^206^ is a specialized loss function extensively used in tasks focused on learning embeddings or conducting similarity-based comparisons. Triplet loss is used to learn embeddings, where similar samples are positioned closer together and dissimilar ones are pushed farther apart in an embedding space. In skin cancer classification, embedding learning via triplet loss could facilitate grouping similar lesions closer and pushing dissimilar ones apart in an embedding space. This process assists in conducting similarity-based analyses, potentially enhancing the model’s proficiency in understanding and classifying skin lesions based on their resemblance. Triplet loss operates by utilizing sets of three data points: an anchor, a positive example (similar class as the anchor), and a negative example (dissimilar class from the anchor). This loss function encourages the model to minimize the distance between the anchor and the positive example, concurrently maximizing the separation between the anchor and the negative example by a predetermined margin. Equation (11) denotes the formula for triplet loss, where *N* represents the number of triplets and *f* denotes the embedding function. $f(x)$ takes $x$ as input and $x^a$, $x^p$ and $x^n$ denote the anchor, positive and negative images, respectively for the $i^{th}$ triplet. $\alpha$ is the bias which acts as a threshold value.

11 $$\begin{aligned} L_{triplet} = \sum _{i=1}^{N} \left[ \Vert f(x_i^a) - f(x_i^p)\Vert _2^2 - \Vert f(x_i^a) - f(x_i^n)\Vert _2^2 + \alpha \right] \end{aligned}$$

### Hybrid loss

Hybrid loss functions involve the integration of multiple individual loss components to create a comprehensive objective function. These hybrid approaches seek to capitalize on the advantages offered by different loss functions, fostering improved model training and performance. For skin cancer classification, various hybrid loss functions have been proposed, designed to tackle distinct challenges, including issues related to class imbalance and feature learning. Le et al.^121^ employed a hybrid loss function, incorporating both weighted loss and focal loss, to evaluate the performance of their skin cancer classification model. Also, Mandal et al.^148^ utilized a hybrid loss function comprising categorical cross-entropy loss and triplet loss to assess the performance of their classification model. Equation (12) represents the hybrid loss function proposed by Mandal et al.^148^, with the scalar weight $w$ determining the relative contribution of the two distinct types of loss functions.

12 $$\begin{aligned} L_{hybrid} = w \times L_{CCE} + (1-w) \times L_{triplet} \end{aligned}$$

## Open challenges

The numerous research methodologies and articles, discussed in this survey, have been successful in addressing many challenges regarding skin cancer classification. Despite promising outcomes, the current methodologies fall short in delivering consistently strong performance within real-world settings. This section outlines the unresolved issues and open challenges that necessitate further attention and exploration by researchers.

1. **Limited data and imbalanced datasets:** DL models require a large amount of labelled data for effective training. However, obtaining a diverse and comprehensive dataset for skin cancer images, with different types, stages, and demographics, can be challenging. Additionally, imbalances in class distribution can lead to biased models that perform poorly on underrepresented classes, thereby hindering the accurate classification of less common types of skin cancer, potentially leading to misdiagnosis or overlooking of critical cases.
2. **Limited annotated data:** Annotating and labelling skin lesion images by experts is time-consuming, expensive, and prone to human error. Developing efficient and reliable methods for annotating data is crucial to facilitate model training.
3. **Inter-class similarity and intra-class variability:** A crucial challenge lies in distinguishing between different skin lesions due to their subtle variations and similarities. Both benign and malignant lesions often share visual characteristics, such as color, texture, shape, and patterns, blurring the distinction between harmless moles and potentially cancerous growths. This similarity between different lesion types (inter-class similarity) presents a significant hurdle for automated classification systems. Moreover, another challenge stems from the variability within the same class of skin lesions (intra-class variability). Even among malignant lesions, there exists considerable diversity in appearance. For example, melanomas can display a broad spectrum of colors, shapes, and patterns, complicating the establishment of clear boundaries between various types of lesions. This intra-class variability adds an additional layer of complexity to the classification process, demanding models to capture and comprehend the subtle differences within each lesion category.
4. **Small lesion detection:** Detecting small and subtle skin lesions, especially in high-resolution images, is an uphill challenge. DL models may fail to identify tiny lesions or produce false negatives due to their size and the presence of various imaging artifacts.
5. **Robustness to variations:** Skin cancer images can exhibit variations due to inconsistent lighting conditions, background noise, camera and image quality. Models need to be robust to such variations to ensure accurate and consistent performance.
6. **Generalization to diverse population:** Models developed on data from specific demographics may lack generalizability across diverse skin colors, ethnicities, ages, or geographical regions. This is particularly important in skin cancer classification where the model should be able to perform accurately on various skin types. Developing models that perform consistently across diverse populations is a significant challenge.
7. **Interpretability and explainability:** Skin cancer classification requires accurate and interpretable predictions to aid medical professionals in making informed decisions. While DL models can achieve high accuracy, they often lack transparency, making it challenging to explain their decisions to clinicians and patients. Interpretability is extremely crucial for trust and acceptance, especially in medical applications. Clinicians need to understand why a model arrived at a particular diagnosis to trust and validate its recommendations. In contrast, traditional ML models offer greater interpretability, allowing clinicians to grasp the underlying decision-making processes more easily. However, these models typically sacrifice accuracy compared to their DL counterparts. Consequently, the challenge lies in striking a balance between performance and interpretability. Clinicians may prefer models that are easier to understand, even at the expense of some predictive power, while data scientists may favor models that deliver higher accuracy but lack transparency. Addressing this trade-off is vital for the successful integration of computer vision-based systems in healthcare, where both precision and trust are paramount.
8. **Computational constraints for real-time diagnosis:** Deploying computer vision-based models for real-time diagnosis of skin cancer in clinical settings requires rapid and accurate assessments, which may be constrained by computational limitations. Delayed or inaccurate diagnosis due to computational constraints can hinder the timely treatment of skin cancer.
9. **Lack of standardization:** While research in skin cancer classification using computer vision is progressing, a notable issue identified during the preparation of this survey is the widespread tendency among researchers to not utilize complete sets of sample images for a particular class, or even entire classes, within their respective datasets. This leads to biased and incomplete evaluations giving rise to unfair comparisons in performance among various existing methods. This lack of a standardized evaluation protocol further complicates the understanding of genuine advancements in state-of-the-art methods.

## Future research directions

Advancements in healthcare, particularly associated with skin cancer classification, could be greatly propelled by addressing the research gaps mentioned in the previous section. Improving the accuracy and robustness of skin cancer classification holds the potential to elevate clinical decision-making, treatment planning, and ultimately, patient outcomes in the field of dermatology. Careful consideration of validation procedures, ethical concerns, and the mitigation of potential biases becomes imperative when introducing novel methods or datasets in the realm of skin cancer classification within the medical community. This section aims to elucidate forthcoming research paths in this domain.

1. **Synthetic data augmentation:** Oversampling techniques or advanced data augmentation techniques that generate realistic variations in lighting, skin tones, and image quality can help alleviate the issues of lack of data and data imbalance. Additionally, GANs can be used to create synthetic skin lesion images, helping to mitigate data scarcity. However, it is essential that these synthetic images not only appear visually plausible but also capture medically significant features. Despite their benefits, using synthetic images in medical diagnosis raises concerns about data authenticity. Healthcare professionals may question the reliability of diagnoses based on artificial images rather than real patient data. To mitigate this, synthetic images should be clearly labelled by medical experts and used alongside real images for validation. It is crucial to emphasize that synthetic images are used with the sole purpose of augmenting limited datasets in order to better model training, not to replace authentic diagnostic data.
2. **Few-shot learning and self-supervised approaches:** Combining transfer learning with few-shot learning or zero-shot learning can help models adapt to new skin lesion categories with limited samples or recognize instances not encountered during training. This approach can leverage knowledge from similar categories while learning quickly from a limited amount of data. Addressing the scarcity of annotated data can also be tackled by employing self-supervised techniques, such as contrastive learning. This approach allows the model to discern the intrinsic structure of features within images. Additionally, achieving a deeper comprehension of meaningful feature representations and continually learning from a data stream can be facilitated through the application of representation learning and continual learning techniques respectively. These strategies contribute to the development of more generalized models capable of adapting to a variety of data scenarios.
3. **Ensemble techniques and deep feature fusion:** Ensemble methods offer a solution to address both inter-class similarity and intra-class variability challenges. Employing diverse CNN architectures within ensembles can mitigate bias and variance errors, consequently enhancing overall accuracy. Meanwhile, deep feature fusion amalgamates multi-level and multi-scale features derived from various layers of base learners, resulting in enriched representations containing diverse information and enhanced discriminative capabilities.
4. **Attention mechanisms:** Attention mechanisms elevate the model’s capability to concentrate on particular image regions, enabling the network to prioritize crucial features while suppressing less significant ones. This selective emphasis contributes to enhanced performance. Additionally, in conjunction with attention mechanisms, feature selection methods can be employed to choose a subset of pertinent features to build more efficient models.
5. **Creation of larger datasets:** Skin cancer classification datasets typically exhibit a limited number of images. Collecting more images and creating larger datasets can facilitate the training of more resilient models capable of generalizing across inconsistent lighting conditions, noise levels, and diverse skin colors, ethnicities, ages, or geographical regions.
6. **Inclusion of diverse images in datasets:** All the standard skin-cancer datasets contain images of fair-skinned people mostly from the USA. To develop a more generalised model with respect to skin color, we need to incorporate images from diverse ethnic groups, varying skin tones and different geographical regions in the datasets which can be used for training purposes.
7. **Explainable AI (XAI):** Developing models that offer explanations for their decisions can enhance transparency and trust. Techniques such as attention maps, saliency maps, and gradient-based attribution can highlight infected regions, aiding in explaining model decisions, and thereby enhancing interpretability.
8. **Real-time diagnosis:** Optimizing models for efficiency, using edge computing, or developing specialized hardware can facilitate real-time diagnosis without compromising accuracy. Edge computing brings computation closer to where the data is generated, allowing for quicker analysis and decision-making, especially beneficial in time-sensitive scenarios such as medical diagnoses.
9. **Collaborative efforts:** Collaboration among researchers, industry experts, radiologists, and dermatologists is crucial. Establishing standardized benchmarks, sharing datasets, and cultivating best practices are key collaborative efforts. Domain-specific insights from medical professionals can validate classification accuracy, offer valuable feedback, and contribute to refining the overall classification process. These collaborations foster the development of more resilient and reliable models.

Undoubtedly, the field of skin cancer classification is poised for substantial evolution in the upcoming years. Enhancing the classification accuracy and clinical applicability hinges on amalgamating state-of-the-art technologies, collaborative efforts, and inventive methodologies. The horizon of this research domain presents an intriguing frontier with the prospect of enhancing medical diagnostics as researchers strive to address existing challenges and delve into uncharted territories.

## Conclusion

Skin cancer classification stands as a pivotal research area, due to its significant impact on global mortality rates. Recent strides in the field showcase the potential of CAD systems for early detection and precise dermatological diagnoses. This review not only presents researchers with insights into the latest developments and gaps in the field, but also offers clinicians practical knowledge on the integration of AI tools for improved diagnostic decision-making. This survey reviews the progression of research trends over the past 18 years, alongside the datasets that have been instrumental in driving these advancements. It encompasses a wide array of computer vision-based studies, ranging from traditional ML techniques paired with handcrafted features to state-of-the-art DL models like CNNs, GANs, and ViTs. Additionally, the survey explores hybrid and multimodal approaches that integrate multiple techniques and data modalities to enhance classification performance. Each paper included in the review is critically analyzed, with notable observations and limitations highlighted to provide a well-rounded understanding of the current state of the field.

Common performance metrics and loss functions used in the literature have been discussed, providing a foundation for evaluating the effectiveness of these models. Key challenges such as data scarcity, subtle variations between benign and malignant lesions, and issues related to model interpretability, have been highlighted. Emerging techniques like attention mechanisms, ensemble learning, few-shot learning, and self-supervised learning hold promise for improving model performance and are outlined as future avenues for research. Addressing the limitations posed by dataset availability, feature complexity, and trust in AI models is crucial for the successful adoption of these systems in clinical settings. The future of skin cancer classification research promises innovation through novel methodologies, domain-specific insights, and interdisciplinary collaboration. As the field progresses, bridging the gap between computer vision-driven CAD systems and clinical practice will be essential, ultimately aiming to improve diagnostic results, treatment planning, and overall patient outcomes in dermatology.

## Data Availability

The datasets analysed in this study are available from the following sources: HAM10000 (https://www.nature.com/articles/sdata2018161), ISIC Archive (https://www.isic-archive.com/), ISIC 2016 challenge (https://challenge.isic-archive.com/data/#2016), ISIC 2017 challenge (https://challenge.isic-archive.com/data/#2017), ISIC 2018 challenge (https://challenge.isic-archive.com/data/#2018), ISIC 2019 challenge (https://challenge.isic-archive.com/data/#2019), ISIC 2020 challenge (https://challenge.isic-archive.com/data/#2020), MED-NODE (https://www.sciencedirect.com/science/article/abs/pii/S0957417415002705), PH2 (https://ieeexplore.ieee.org/document/6610779), DermIS (https://www.dermis.net/dermisroot/en/home/index.htm), DermQuest (https://tspace.library.utoronto.ca/bitstream/1807/47896/1/dv07048.pdf), Dermnet (https://dermnet.com/), Dermofit Image Library (https://licensing.edinburgh-innovations.ed.ac.uk/product/dermofit-image-library).
